# Advanced encryption schemes in multi-tier heterogeneous internet of things: taxonomy, capabilities, and objectives

**DOI:** 10.1007/s11227-022-04586-1

**Published:** 2022-06-13

**Authors:** Mahdi R. Alagheband, Atefeh Mashatan

**Affiliations:** Cybersecurity Research Lab, Toronto Metropolitan University, Toronto, Canada

**Keywords:** Cryptosystems, Internet of things, Privacy preserving, Blockchain, Confidentiality, Security

## Abstract

The Internet of Things (IoT) is increasingly becoming widespread in different areas such as healthcare, transportation, and manufacturing. IoT networks comprise many diverse entities, including smart small devices for capturing sensitive information, which may be attainable targets for malicious parties. Thus security and privacy are of utmost importance. To protect the confidentiality of data handled by IoT devices, conventional cryptographic primitives have generally been used in various IoT security solutions. While these primitives provide just an acceptable level of security, they typically neither preserve privacy nor support advanced functionalities. Also, they overly count on trusted third parties because of some limitations by design. This multidisciplinary survey paper connects the dots and explains how some advanced cryptosystems can achieve ambitious goals. We begin by describing a multi-tiered heterogeneous IoT architecture that supports the cloud, edge, fog, and blockchain technologies and assumptions and capabilities for each layer. We then elucidate advanced encryption primitives, namely wildcarded, break-glass, proxy re-encryption, and registration-based encryption schemes, as well as IoT-friendly cryptographic accumulators. Our paper illustrates how they can augment the features mentioned above while simultaneously satisfying the architectural IoT requirements. We provide comparison tables and diverse IoT-based use cases for each advanced cryptosystem as well as a guideline for selecting the best one in different scenarios and depict how they can be integrated.

## Introduction

The Internet of Things (IoT) is revolutionizing our lives through autonomous communication among everyday objects, facilitating ubiquitous computing, and the transmission of sensitive information. According to the Statistica report, 75.44 billion devices will be connected worldwide by 2025, a 2.5 times increase in 5 years from 2020 [1]. In addition, forecasts expect that the global market for IoT will grow to 1.6 trillion USD by 2025, almost eight times more than the 2020 revenue [2].

IoT networks are rapidly growing due to advancements in communication and networking technologies. Therefore, a comprehensive IoT architecture must integrate diverse technologies, including the cloud computing, edge computing, fog computing, and blockchain. They cooperate to acquire, aggregate, transmit and store large amounts of data [3]. End-point devices (e.g., sensors, actuators, smart meters, smartwatches) generate a massive amount of data and send them to higher-tier systems for storing and processing. In this workflow, security is of utmost importance.

While there has been a lot of research trying to address the security and privacy issues of IoT [4], there remains a variety of challenges that need to be addressed. Makhdoom et al. recently highlighted the most known threats at various layers of IoT systems. They mentioned that data confidentiality as a fundamental feature for IoT systems could mitigate many vulnerabilities [5]. Confidentiality ensures that unauthorized entities cannot access data either at rest or in motion [6]. However, to achieve confidentiality, most of the data-driven IoT security solutions only implement widely-used conventional cryptographic primitives such as RSA, Elliptic Curve Cryptography (ECC), Identity-Based Encryption (IBE), and ElGamal as surveyed in [3, 7, 15]. While these conventional encryption functions provide confidentiality when appropriately implemented, they do not offer additional features, such as privacy-preserving, resistance to a single point of failure, and malicious behavior detection. Also, they are most demanding and work based on some prerequisites, such as trusted third parties in the setup phase.

***A. Motivation***

The evolutionary development of IoT security solutions is in need of new primitives with greater functionalities, and secure characteristics [28]. This is because only the prevalent signature and encryption primitives are used in practice. Currently, a gap exists between cryptography research and adoption in practice. Therefore, it is imperative that we implement the state-of-the-art primitives with more sophisticated functionalities and less prerequisites [29]. Shai Halevi describes the state-of-the-art cryptographic primitives with three features: they have new functionalities that were needed, they are fast enough to be useful, but they have not reached a necessary level of usability for them to be put into practice [30].

There are a handful of state-of-the-art encryption schemes that provide more secure characteristics such as privacy-preserving, forward security, key-escrow-free, and working without any Trusted Third Party (TTP) entity to mitigate some challenges in IoT platforms. In this paper, we delve into four of these advanced cryptographic primitives that provide confidentiality and discuss how they can be integrated with an IoT architecture that can leverage the said functionalities effectively.

***B. Current problems***

The classical and conventional cryptosystems used marginally meet the same standard of some prominent IoT features due to intrinsic weakness by design. First, their initialization assumptions might a conflict with recent secure IoT demands. Second, the conventional ones should be performed repeatedly for some applications, which considerably increases computation and communication costs. Thus we focus on some recent cryptosystems in this paper which accomplish some of the following characteristics [6, 7, 31].

**1)** ***Trust Management.*** The proliferation of numerous technologies in complex advanced IoT architectures needs a new model of security assumption to know as *zero trust*. It is a collection of concepts and ideas to brace the least privilege principle and utilize zero trust concepts [32]. Although it is an end-to-end approach and encompasses all aspects of cybersecurity, we intend to clarify this point of view in cryptographic primitives.

Reliable and trustworthy entities are prerequisites of many security mechanisms. For instance, Trusted Third Party (TTP) is used in the key distribution phase in ECC-based, RSA-variant, and most identity-based encryption schemes. A trusted entity should select a few prime numbers, unified elliptic curve equation, master key, etc. In practice, having such entities are problematic. The less trust an algorithm takes on other entities, the more appropriate it is for IoT-oriented applications; implementing a standard-based zero trust architecture is demanding [33].

2) ***Functionalities.*** Apart from only encryption and decryption for supporting confidentiality in legacy systems, as Halevi mentioned [30], some supplementary IoT-friendly functionalities can be provided with advanced cryptosystems.

- First, privacy-preserving features are essential for most parts of IoT systems. Due to the massive scale of IoT, privacy issues have remained a significant challenge. It is typically regarded as different notions, including anonymity, unlinkability, untraceability, and forward security in various applications.
- Second, alignment with IoT architecture is of utmost importance. For instance, interoperability between two deployed networks is an additional functionality that is not supported by conventional cryptosystems. Two different IoT networks with diverse cryptographic assumptions should be able to interact and share data. There are some advanced cryptosystems that can bridge them with conversion mechanisms.
- Third, Single-Point-of-Failure (SPoF) avoidance is another instantiation. SPoF stops the entire system from working if a failure happens. Therefore, designing cryptosystems with no SPOF is highly desirable. Hierarchical IoT networks are more vulnerable to SPoF because an entity on top of the hierarchical structure controls the objects within the network. Some advanced cryptosystems do not require a trusted entity and assist IoT to resist SPOF.
- Fourth, some IoT-based applications require high availability, which is ability of systems to operate perpetually without stopping. Some advanced encryption schemes can aid in distributing data and clustering to prioritize availability. Not that working without SPOF is an instantiation of high availability. Also, scalability, which is the ability of IoT nodes to adapt to changes in the network topology after deployment, is an important attribute in designing cryptosystems.

**Fig. 1:**
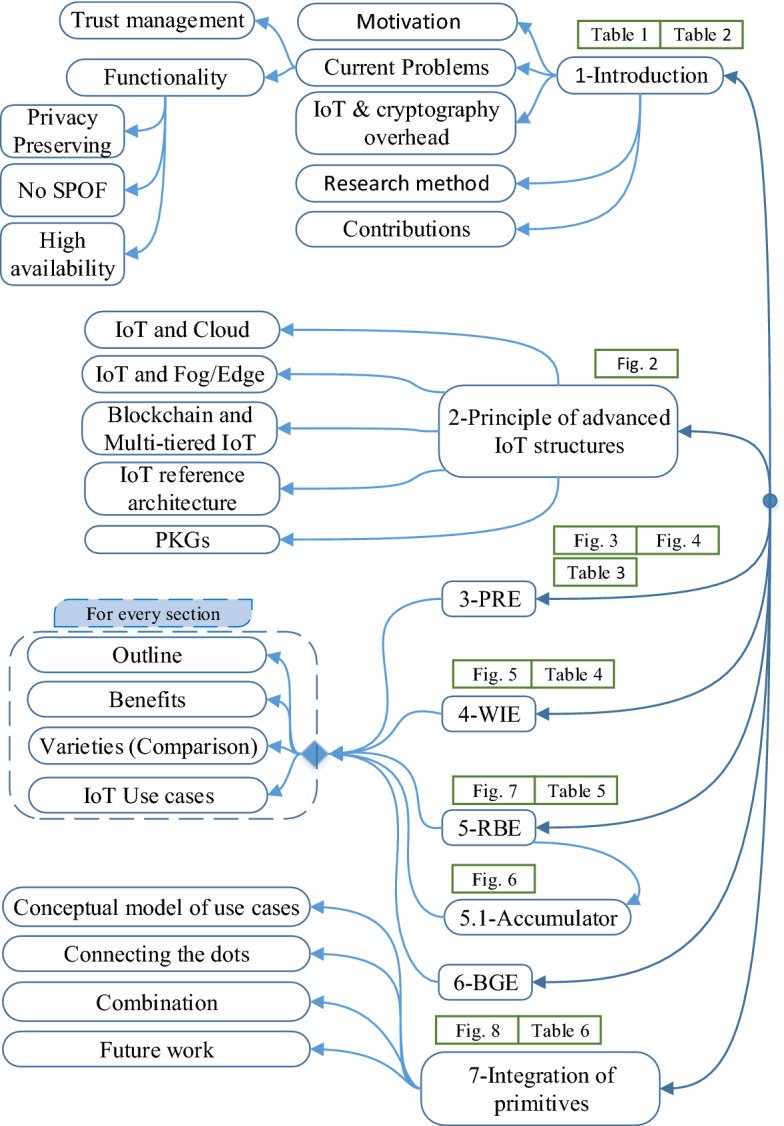
The organization of this survey paper (Sections 3 up to 6 have a similar structure)

**Table 1:** The comparison of recent survey articles on security and privacy of IoT with data encryption perspective

IoT security survey papers	Covered technologies	1	2	3	4	5	6	7	8	9	10	11	12	13	14	15
This paper, 2022	P2P, Edge/Fog, Cloud, Blockchain	$\checkmark$	$\checkmark$	$\checkmark$	$\checkmark$	$\checkmark$	–	-	–	–	–	–	–	–	–	–
Zhang [8], 2018	P2P, Edge/Fog	–	$\checkmark$	–	–	–	$\checkmark$	–	$\checkmark$	–	–	–	–	–	–	–
Wang [9], 2019	Blockchain	–	–	–	–	$\checkmark$	–	–	–	–	–	–	$\checkmark$	–	–	–
Shrestha [10], 2019	Blockchain	–	–	–	–	–	$\checkmark$	–	–	–	–	–	–	–	–	–
Harbi [11], 2021	Fog, Edge, Blockchain	–	–	–	–	–	$\checkmark$	$\checkmark$	$\checkmark$	–	–	–	–	–	–	–
Mousavi [12], 2021	Cloud	–	–	–	–	–	–	$\checkmark$	$\checkmark$	–	–	$\checkmark$	–	–	–	–
Raikwar [13], 2019	Blockchain	–	–	–	–	–	–	–	–	$\checkmark$	$\checkmark$	–	–	–	–	$\checkmark$
Noor [3], 2019	Cloud, Blockchain	–	–	–	–	–	–	$\checkmark$	–	–	–	$\checkmark$	–	–	–	–
Sfar [14], 2018	Edge	–	–	–	–	–	–	$\checkmark$	–	–	–	$\checkmark$	$\checkmark$	–	–	–
Kouicem [7], 2018	P2P	–	–	–	–	–	–	$\checkmark$	$\checkmark$	–	–	$\checkmark$	–	$\checkmark$	–	–
Malik [15], 2019	P2P	–	–	–	–	–	–	$\checkmark$	–	$\checkmark$	–	–	–	–	–	–
Yang [19], 2017	P2P	–	–	–	–	–	–	$\checkmark$	-	$\checkmark$	–	–	–	–	–	–
Lohachab [16], 2020 Fernand. [20], 2019	P2P	–	–	–	–	–	–	–	–	–	–	–	–	–	$\checkmark$	–

(Enc.: encryption, IBE: Identity-Based Encryption, PKC: Public Key Cryptography, P2P: Peer-to-Peer) (The encryption algorithms: 1—Registration-based Encryption, 2—Proxy Re-Encryption, 3—Wildcarded Encryption, 4—Break-Glass Encryption, 5—Cryptographic Accumulator, 6—Homomorphic Encryption, 7—Conventional public key encryption (RSA, ECC), 8—Attribute-Based Encryption (ABE), 9—Identity-Based Encryption (IBE), 10—Broadcast Encryption, 11—Symmetric Encryption, 12—Hash functions, 13—Signcrption, 14—Post-quantum Encryption, 15—Incremental Encryption)

This paper is a stepping stone to bring new cryptographic advancements into IoT-driven applications and mitigate the mentioned challenges. The chosen primitives provide a noticeable new viewpoint on related research communities. The amalgamation of state-of-the-art primitives and current security-supporting solutions can increasingly strengthen IoT systems. In response, the new solutions can be used in IoT-oriented technologies, like blockchain, cloud, fog, and their applications, such as smart contracts, data aggregation, access control, etc.

***C. IoT and Cryptographic Primitives Cost***

IoT systems comprise a broad range of technologies. Bluetooth, LR-WPAN and Z-WAVE technologies are examples of low-cost solutions for data transmission in the physical layer of IoT edge devices. Wi-Fi, cellular communication (4G, 5G), and LoRa have medium communication and computation cost [17]. Optical fiber communication can be applied among fogs and clouds servers [18]. On average, IoT-supported hardware has about 285MB memory and 330 MHz clock speed [18], which is sufficient for the execution of asymmetric cryptography primitives.

Moreover, we require derivatives of asymmetric public key encryption algorithms for IoT systems. According to the advancements in IoT hardware devices, they can bear the burden of asymmetric-variant of encryption algorithms. Some instances are explained as follows.

- Rahulamathavan et al. used heavy attribute-based encryption for privacy-preserving in IoT [21]. Also, an attribute revocation system is simulated for access control in IoT platforms, [22].
- Zhou et al. implemented Fully Homomorphic Encryption (FHE) in a blockchain-enabled IoT system for outsourcing computation [23].
- Many IoT-based authentication and bootstrapping protocols, such as Diffie-Hellman Key Exchange (DHKE), Datagram Transport Layer Security (DTLS), have been proposed in the context of digital certificate and Public Key Infrastructure (PKI) [15].
- There are a few underlying cloud-based IoT platforms which support distributed computing and various communication protocols including AWS IoT from Amazon, ARM Bed from ARM and other partners, Azure IoT Suite from Microsoft, Brillo/Weave from Google, Calvin from Ericsson, HomeKit from Apple, Kura from Eclipse, and SmartThings from Samsung. Their devices mostly support PKI [24].

*Research method*. We took the following steps for writing this survey paper to connect the advanced cryptosystems. First, we considered the prominent IoT architectures and introduced a comprehensive one, including the most critical IoT-driven technologies. This architecture is the building block of the parts of our research. Second, we investigated the state-of-the-art cryptographic primitives in related top conferences and journals and how they can play a significant role in IoT security and privacy solutions to mitigate trust management challenges and increase I0T-driven functionalities.

Note that we merely focus on the cryptographic primitives that have not been considered in the IoT context. We highlighted the suitable ones that have been surveyed before in Table 1, and we excluded them in this paper. For instance, Homomorphic cryptosystems are one of the promising IoT-driven encryption methods that have been repeatedly surveyed and are mentioned in Table 1. The partially, somewhat, and fully Homomorphic encryption schemes are secure and privacy-preserving methods that allow a blockchain or cloud server to compute some operations on encrypted data [25]. The authors of [8] discussed and compared the steps of prevalent homomorphic encryption mechanisms. Shrestha and Kim highlighted many use cases for the integration of IoT, blockchain, and homomorphic encryption [10]. Recently, Harbi et al. reviewed homomorphic encryption for cloud, fog, and edge computing in IoT [11]. Additionally, merging network coding and homomorphic cryptosystems can reduce latency and increase network reliability [26]. Aulakh and Ramachandran carried out a recent survey on fully homomorphic encryption standards for IoT and cloud computing [27]. The mentioned papers can be a stepping-stone toward using homomorphic encryption in IoT systems.

Similarly, there is a noticeable quantity of research on attribute-based encryption [7, 8, 11, 12] and identity-based encryption [13, 15, 19] in IoT. However, some advanced varients of ABE and IBE will be discussed in this paper.

We examined more than 30 newly proposed concepts and selected five advanced IoT-friendly primitives. To the best of our knowledge, our work is the first to close the research gap between the latest advancements in cryptography and multi-tiered IoT networks to solve the real problems of IoT applications.

***D. Our Contributions***

The main contributions of this paper are fourfold:

- Discuss the technologies applied in various IoT platforms, including cloud, fog, edge, and blockchain technologies, and highlight their advantages and drawbacks in IoT.
- We design a comprehensive and multi-tiered IoT reference architecture that covers all technologies and elaborates on their interaction. This architecture is the basis of this paper’s contributions.
- Survey advanced cryptography primitives. We focus on state-of-the-art cryptographic primitives. Most of the main primitives in this paper have been published since 2018. We elaborate on the unique characteristics of each cryptographic primitive and emphasize how they can be used in the IoT infrastructure. It should be pointed out that we focus on cryptographic primitives and do not discuss security protocols. The considered primitives are as follows:
  - Proxy Re-Encryption (PRE)
  - Wildcarded Identity-based Encryption (WIE)
  - Cryptographic accumulator as a prerequisite (CAC)
  - Registered-Based Encryption (RBE)
  - Break-Glass Encryption (BGE)
- We provide the taxonomy of the mentioned primitives and show their relationships. It is an in-depth guide for choosing the appropriate ones in IoT networks with different assumptions and security requirements.

**Table 2:** The list of acronyms

Acronym	Definition
EFCB	Edge-Fog-Cloud-Blockchain
*pp*	Public parameters
*PU*	Public key
*Pr*	Private key
PRE	Proxy re-encryption
CP-APRE	Ciphertext policy attribute-based PRE
TTP	Trusted third party
KP-APRE	Key policy attribute-based PRE
PKG	Private key generator
PPRE	Puncturable PRE
IBE	Identity-based encryption
BPRE	Broadcast PRE
WIE	Wildcarded identity-based encryption
HPRE	Hybrid PRE
DIBE	Downgradable IBE
RBE	Registered-based encryption
BE	Broadcast encryption
PKA	Public key accumulator
BGE	Break-glass encryption
SXDH	Symmetric eXtended Diffie–Hellman
Acc	Accumulator
SPoF	Single point of failure
PKI	Public key infrastructure
TTP	Trusted third party

In recent years, multiple security and privacy aspects of IoT varying degrees of depth, and different scopes were surveyed. Table 1 illustrates a comparison of our research outlining the most-cited and recently published data-driven surveys on IoT security. As shown in Table 1, the state-of-the-art primitives discussed in this paper are distinct from the other conventional solutions. The list of acronyms used in this paper is summarized in Table 2. We also included Fig. 1, a flowchart of the organization of this paper, to promote reader accessibility and ease.

The remainder of this paper is organized as follows. Section 2 describes the paradigms of multi-tiered IoT networks. Sections 3 and 4 elaborate proxy re-encryption, wildcarded and downgradable encryption respectively. Section 5 discusses RBE and cryptographic accumulator as its prerequisite. Then Sect. 6 delves into BGE schemes.

After defining the IoT reference architecture, sections two to six exemplify a unified format to keep this paper highly readable. After simply explanation in “*Outline*” portion, the “*Benefits*” part elaborates on the advantages of every cryptosystem for IoT. We then compare the different types of the corresponding cryptosystem in the “*Varieties*” part. The “*IoT Use cases*” part elaborates on the practical scenarios for applying the corresponding cryptosystem into the IoT reference architecture. Finally, Sect. 7 depicts the integration of primitives for distinct assumptions and applications, followed by the conclusion.

## An overarching IoT reference model

In this section, we discuss the related technologies and orchestrate them to design the IoT reference architecture. The architectural framework of IoT is still not mature in industries and academia. The lack of a widespread structure delays the standardization process and hinders the global adoption of IoT [34]. Blockchain, cloud, fog, and edge paradigm architectures can fill the technological gap, and with high efficiency and back heterogeneity, and hierarchical structures. This reference architecture model helps to justify the necessity of the new cryptographic primitives. Each part of the designed reference architecture in this section represents distinct characteristics and can be partly applied to specific applications. For the most part, IoT platforms have the following intrinsic features:

- ***Heterogeneity***: IoT is an exemplary instance of heterogeneity. IoT encompasses various participant elements, including various lightweight nodes as well as more resourceful entities to manage edge computing, fog computing, cloud computing, centralized storage, and blockchain services [35]. Nodes are embedded devices such as smartwatches, vehicles, appliances, sensors, smart meters, cameras, and wearable devices. Edge devices are mostly smartphones and laptops. Fog entities are base stations, mini servers, gateways, while the cloud services are sophisticated storage and servers.
- ***Hierarchy***: The multi-layered structure of IoT aids us to coordinate the heterogeneous computing and storage paradigms. This approach supports the next-generation services with high bandwidth and low latency [36]. Additionally, hierarchical network models implicitly back Software Defined Network (SDN) to remotely manage intermediary network devices of IoT [37].

In the following text, different paradigms applied in IoT for massive interconnection is elaborated.

### A. IoT and Cloud

The cooperation of cloud services and numerous IoT nodes is very effective. The cloud stores and manages the massive amount of data flow generated by nodes [28]. However, some drawbacks have been reported in this centralized model:

- The cloud services are considered by some critics as the root of privacy violation, which they call the cloud-based IoT “*Internet of Fails*” [38].
- Scalability in IoT networks is another challenging issue for cloud services. Linear growth of cloud resources cannot meet the exponential increase of data production by IoT nodes [8].
- Unpredictable latency for real-time IoT applications causes adverse impacts on availability [39].

Therefore, using only a cloud server for many connecting embedded devices might have several disadvantages. It is necessary to partly delegate storing and processing of data overheads to some intermediary devices.

### B. IoT and Fog/Edge

The concept of fog and edge technologies is rooted in cloud computing, but they are used in the lower tiers of IoT networks. Cloud servers cannot properly offload real-time applications because of latency issues, constrained bandwidth, network congestion, distance, and jitter. Thus, nodes require some intermediaries.

These technologies have many advantages for IoT networks. Not only can fog/edge computing complement cloud services by locally storing, computing, and aggregating data, but they also make IoT networks more distributed and secure [8]. Fog/Edge entities minimize network congestion and latency, tackle connectivity bottlenecks, enhance scalability, back heterogeneity as well as location-awareness, offload computation and promote decentralization [40, 41]. They also facilitate self-adaptive dew computing based on the low-end devices in hierarchy heterogeneous IoT networks [42]. Dew computing is a new complementary piece of cloud computing. Dew computing is the ground level of cloud/fog computing paradigms in a vertically hierarchical structure to distribute the workload of micro-services [43, 44].

#### Fog versus edge

Although fog and edge paradigms are used interchangeably in some papers [45], there are some slight differences between edge and fog services. Edge computing partly carries out fog’s responsibility.

- First, edge devices are distributed and support mobility. Mobile edge computing is one of the most highlighted applications performed at the edge of networks [46]. They noticeably improve system performance and reduce response time [47].
- Second, fog devices generally are cloudlets, mini-servers, or base stations, but the edge layer mostly includes commonplace devices such as laptops and smartphones [48].
- Third, unlike fog devices that are not necessarily at the edge of IoT networks, edge devices are the first contact device with IoT embedded devices (nodes). In fact, both edge and fog services are close to nodes; however, the edge is in the one-hop distance with nodes, while fog devices are a few hops away from nodes.
- Fourth, edge paradigms are more node-focused, but fog paradigms are more infrastructure-focused. Edge devices are at the edge of IoT networks and fog devices are located at the edge of infrastructure [40]. Edge devices are similar to a local gateway and provide computing and storage resources for nodes in the same LAN and cooperate with their counterparts [49].

Despite the slight discrepancies, the functionality of the two terms is almost similar. Fog and edge paradigms are congruent and emphasize the hierarchical and heterogeneous nature of IoT architectures. Both edge and fog play the role of an aggregator and considerably reduce the huge amount of communication bandwidth required to accumulate the nodes’ data.

#### Cloud and fog/edge cooperation

Although fog services can be used as stand-alone services, there is a synergistic effect when a cloud-fog framework is applied. This effective cooperation is one of the promising IoT structures [50–52]. The most noticeable benefits of this structure are as follows.

- This structure provides more computational and storing capabilities in collaboration with cloud services. Fog computing in collaboration with cloud platforms reduces the computational cost by almost 40% [53].
- A wide variety of communication technologies can be applied in cloud-fog-edge architecture, ranging from RFID, Bluetooth, and NFC for short distances to WiFi and LTE-Advanced for long distances.
- A fog-and-cloud-assisted IoT architecture provides a range of new services such as smart infrastructure management and time-sensitive applications with faster real-time response [54]. In the healthcare system, a fog-cloud IoT platform is proposed for monitoring of COVID-19 outbreak [55].
- It manages locally dispersed nodes in a very large scale of networks and covers interoperability [34]. This combination promises better-localized accuracy for IoT-based applications [56].

Briefly, the hierarchical topology of Cloud-Fog-Edge IoT architecture is becoming a dominant structure [40, 57]. However, there is still the challenging issue that nodes have to place their trust in the cloud, fog, and edge entities. In the next section, we aim to mitigate this problem with the aid of blockchain technology.

### C. Convergence of blockchain and multi-tiered IoT

Blockchain is a type of distributed ledger technology composed of a sequence of blocks linked by hash digests [58]. The notion of blockchain was introduced by Haber and Stornetta in 1990 [59] and then became popular when implemented as a cornerstone of Bitcoin in 2008. Blockchain is an emerging technology that improves IoT networks’ transparency, reliability, and efficiency. Blockchain orchestrates the combination of multiple technologies to provide immutability, integrity, traceability, and pseudonymity through distributed ledgers [60]. The real-time data provided by nodes can be stored in a blockchain using decentralized and distributed ledgers. There are plenty of papers that discuss blockchain applications. For instance, the authors of [61] surveyed applications not related to cryptocurrencies such as identity management, access control, and records management. Privacy-preserving and trust management can be backed by blockchain-based solution in dynamic networks with high mobility [62]. Syed et al. proved that using blockchain in IoT can remarkably decrease cost and scalability constraints with more reliability [63].

#### Blockchain and IoT interaction

There are some reasons that blockchain technology is becoming increasingly prevalent in IoT networks. First, privacy invasion is an intrinsic threat in cloud-based and fog-based IoT networks, despite their benefits, because all nodes have to trust cloud and fog. The so-called TTPs might unscrupulously use the users’ sensitive information. The evidence like PRISM project as a data surveillance program [64] confirms that this issue may be happened. Thus, many users have misplaced their trust in so-called trusted third parties [65]. In contrast, blockchain can provide reliable peer-to-peer connections over an unreliable IoT network without any TTP [60]. Second, IoT networks guarantee the accountability of participant nodes based on blockchain’s immutability [34]. Third, anonymity and untraceability of sensitive data that are necessary for some applications are provided to some extent by blockchain [66].

Moreover, the following features indicate that blockchain technology improves the efficiency of IoT networks: a) the elimination of centralization and SPoF which improves fault tolerance; b) playing the role of a proxy server [67, 68]; c) decreasing the heavy load on cloud /edge/fog entities and reducing many-to-one traffic flows; d) increasing network scalability and programmability because all nodes fairly provide resources for cooperation [60]; e) providing a reliable incentive scheme to encourage participants; and f) reducing maintenance costs compared to centralized cloud services. For example, the cost of using Sia, a blockchain-based storage platform that uses a peer-to-peer network [69], is less than 10% of using Amazon AWS cloud computing platform [34].

To indicate that blockchain-based IoT networks are practical, we explained two implemented instances. First, IOTA is a promising example of a blockchain-oriented IoT solution. IOTA is an open-source, permissionless distributed ledger especially designed for IoT devices. It is possible to securely store data within transactions or even spread larger amounts of data across multiple bundled transactions. The IOTA structure is based on a directed acyclic graph for storing data for node-to-node interactions. IOTA has an acceptable level of security to be used at the device’s middle servers [70, 71].

Second, lightweight cryptocurrencies [72] offer the potential to incentivize many nodes to participate in data transactions. Blockchain-driven IoT nodes control themselves. Moreover, the decentralized data storage management keeps data completely private through a blockchain that manages access controls and stores logs of events [73]. This management system ensures nodes that all violations of access policies are detectable without any TTP server, and the data is stored with an off-chain storage solution [74]. Third, blockchain mixing protocols and pseudonymity solutions considerably anonymize the participant nodes. Also, designing a blockchain-based IoT authentication framework (e.g. [75]) is a current research trend. Digital forensic in IoT can be investigated by making the chain of custody on blockchain [76].

#### Blockchain types

There are three different types of blockchains based on group policy nodes. *a*) Public or permissionless blockchains allow everyone to store a copy or validate new blocks. *b*) Private or permissioned blockchains are ones where every node should be recognized before joining the blockchain. It is an applicable solution to prevent malicious data modification and trace data exchanges between nodes. It is much lighter than permissionless blockchain requiring no processing fee or consensus routines. For example, Ripple and Hyperledger are permissioned blockchain instances that work with IoT. The blockchain concept, particularly permissioned ones, improves the throughput of IoT interactions. *c*) Consortium or federated blockchains are permissioned to keep reliability and transparency among the involved clusters [34].

#### Blockchain-IoT realization

There are three major methods to store data in a block-chain: sidechain, sharding, and directed acyclic graph.

- **Sidechain** is a peripheral blockchain attached to the parent blockchain and stores the less critical digital assets. The two blockchains interchangeably transfer the required data. Sidechains increase flexibility, scalability, and reduce the traffic on the parent blockchain [77]. Sidechains are synchronized with the parent blockchain [34]. The main blockchain can be a public blockchain controlled by users on the Internet. It is a developing method, particularly in public and private consortium blockchains such as Liquid [78]. Some networks can connect a few independent blockchains with this approach. For example, COSMOS is a high-level blockchain that interconnects some parallel blockchains to interoperate with each other [79]. Singh et al. reviewed and compared many sidechain platforms [80].
- **Sharding** is a technique that divides a parent blockchain into several sub-chains (shards) to improve performance, reduce response time, and overcome scalability matters. Nodes would then be assigned to individual sub-chains and communicate in parallel at the same time [60, 81].
  Furthermore, this mechanism is genuinely compatible with IoT structures and can modify interoperability because each shard can be used for a group of distinct nodes or intermediary servers in a specific zone. The sharding technique can split the overhead of processing among a smaller group of nodes that results in higher throughput and lower latency [82].
- **Directed Acyclic Graph** (DAG) technique is a lightweight and IoT-led mechanism. Since DAG supports different kinds of transactions and chains, it is suitable for large and heterogeneous networks. There are some IoT-oriented applications such as IoTchain [83], B-IoT [84], and IOTA [70] that apply DAG.

Overall, since the mentioned techniques remarkably reduce the computation and communication overheads, they can assist blockchain technology to be adopted with IoT platforms.

#### Blockchain and cloud/fog/edge alliance

There is a synergy between using blockchain and fog /cloud entities. This combination mitigates many drawbacks, consumes fewer resources, and helps users to benefit from the advantage of decentralized and centralized entities. The two aspects are discussed as follows.

(a) On the one hand, blockchain technology contributes to threat mitigation. Some potential threats might cause intentional or unintentional security breaches when using the centralized services (Cloud/Fog/Edge). The lack of enough transparency and the need for trust justify using blockchain in parallel with centralized entities.

To give some instances, Liang et al. used blockchain to aid cloud and fog paradigms for data provenance [85]. Xie et al. discussed the benefits of using blockchain for provisioning and managing connections among multiple cloud services [86]. Rathore et al. employed blockchain-cloud services to save power consumption. They evaluated the performance of decentralized blockchain-based architecture and demonstrated that the computational overhead of this structure is almost 25% less then having only centralized and distributed architectures [36].

In addition, Qiu recently proposed a cloud mining approach in blockchain-based IoT networks and offloaded mining tasks to cloud servers [87]. Gai et al. used a permissioned blockchain for privacy preservation in edge computing [88]. A method of combining different computing and blockchain technologies addressed privacy and security issues in their model. Moreover, smart contracts accompanied by the blockchain technology achieved an optimal resource allocation [88]. Jeong et al. used blockchain to protect users’ privacy in a cloud-based hierarchical IoT environment. Users’ identifiable information is classified into a blockchain to prevent malicious use in the cloud environment [89]. Recently, Blockchain-as-a-Service (BaaS) platforms have been developed as a promising solution to increase productivity. The BaaS framework provides blockchain service over cloud computing [90, 91]. A BaaS can also take advantage of smart contract to receive data from IoT nodes [92].

(b) On the other hand, cloud and fog paradigms contribute to blockchain technology. Since most nodes of IoT are subject to resource constraint and cannot store a copy of blockchain or validate blocks, edge and fog servers play the role of ledgers [34]. There are three solutions to connect nodes and blockchain. The following approaches can be applied together at the same time in different layers depends.

(i) Connection of nodes to the blockchain via the edge and fog devices as gateway devices: edge and fog entities store the aggregated data in a sidechain. This method is somewhat decentralized and nodes have no direct connection with blockchain [93, 94].
(ii) All nodes directly integrate with blockchain: this approach is the fastest solution. This approach would have increased computational and communication overheads with more autonomy [34, 95].
(iii) Hybrid cloud-blockchain approach with fog cooperation: nodes have a choice to send messages to the cloud, fog/cloud, or blockchain, and the fingerprint of all messages is stored in a blockchain. All technologies cooperate in this hybrid approach to overcome the limitation of both centralized resources and blockchain IoT networks [95]. The schemes in [96–98] are a few noticeable hybrid cloud-blockchain IoT-based systems.

Overall, *Multi-tiered IoT+Blockchain* is a promising architecture. Apart from reducing costs, its intrinsic characteristics expedite designing of solid security-oriented protocols [100]. However, this collaboration is still in its infancy. In the next section, we plan a comprehensive model driven by the discussed paradigms.

### D. IoT reference architecture

**Fig. 2:**
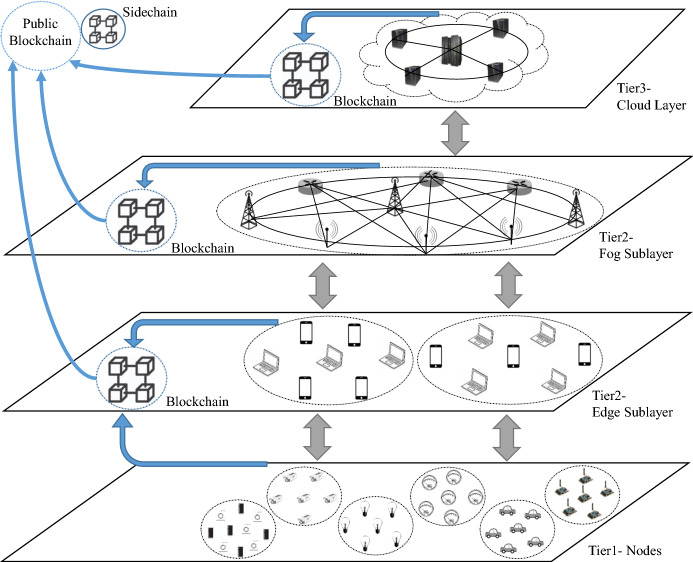
IoT reference architecture (EFCB-IoT). The synthesis of Edge, Fog, Cloud, and Blockchain technologies with Peer-2-Peer IoT nodes

As we mentioned, there is no generally accepted IoT architecture and each one has some positives and negatives. However, according to the thorough discussion and mentioned compelling reasons, we design an IoT reference architecture called Edge-Fog-Cloud-Blockchain-IoT (EFCB-IoT) architecture, which is aligned with diverse applications. This model hits two birds with one stone. Not only does EFCB-IoT benefit from the edge, fog, and cloud paradigms, but also blockchain proactively protects them from insider and outsider malicious activities and enhances the quality of service.

Figure 2 depicts the EFCB-IoT architecture in three tiers. Tier 1 is comprised of many groups of heterogeneous nodes which have peer-to-peer connections together. Also, they cooperate with the hierarchical cluster-based connections to send the generated data to the corresponding edge devices. IoT nodes comprise wide variety of devices such as embedded microchips, smart gadgets, and sensors. They might be connected to humans and vehicles or operate as stand-alone devices to generate data and transfer to higher tiers and a local blockchain for data validation. Also, they can communicate with neighboring devices. tier 2, as an intermediary layer, includes two sub-layers allocated for edge and fog services. As we discussed, the mediating levels collect and aggregate the delivered data to higher entities for storing and more analysis. The local, fine-tune, and permissioned blockchain in this tier keep a fingerprint of all interactions to back data assurance. The cloud servers are located in the tier 3. The cloud securely stores all data for future retrieving, analysis, data warehousing, and computing. The cloud layer is supposed to be a Trusted Third Party (TTP), which is monitored by the local blockchain.

The EFCB-IoT model is an appropriate combination of centralization and decentralization. The public blockchain is, in fact, a public accumulator for data integrity verification in the three tiers. It prevents malicious edge, fog, or cloud paradigms from changing stored messages by keeping the summary of all interactions in the public blockchain. It means that using blockchain in EFCB-IoT architecture is a supporting layer that prevents cloud-fog-edge misbehavior and provides immutability. The public blockchain, which can be monitored by all nodes and external entities, stores the digest of all records executed by the cloud, fog, and edge layers. It provides data provenance for the collected data in various use cases. Unlike the local blockchains in the tier 2 and tier 3 which are permissioned and private, the entities out of the IoT framework like public ledgers can participate and perform monitoring. Moreover, as we discussed earlier, the sidechain is a child blockchain which takes loads off the parent blockchain by storing some less important data. This collaboration aids to reduce computation and storage cost of the public blockchain.

### E. Accountable and auditable private key generators

Although key distribution is out of the scope of this paper, it is a crucial prerequisite for some primitives discussed in the rest of this paper. Most encryption and digital signature primitives are driven by a Private Key Generator (PKG) as a TTP entity, so the reliability of PKGs are essential. However, it is not always achievable. There are public-key encryption methods, such as IBE, that suffer from the inherent key escrow problem because a fully trusted PKG can decrypt all ciphertexts of every node. Using multiple PKGs to collaborate in generating master private keys mitigates the vulnerabilities, but sacrifices the accountability of each PKG. PKGs can collude to generate and deliver up nodes’ private key. The following solutions are based on the concept of decentralized PKG to alleviate the dominance of PKG and reduce their possible misbehavior.

- Recently Zhao et al. added accountability to distributed PKGs’ solutions in such a way that the traitor PKGs are traceable [99]. The Edge/Fog para digms can play the role of distributed PKGs in multi-tiered IoT systems, allowing every node to recognize the identity of dishonest PKGs. Fujioka et al. formally considered the relation between security notions of PKG-based and distributed PKG-based systems. They proposed general constructions of IBE based on multiple PKGs [101].
- Auditable private key generation for joining, generating, and verifying keys is another solution achieved by blockchain technology. They used distributed ledger and consensus techniques to achieve auditability and verification in key generation [102].

In the rest of this paper, we discuss four advanced encryption schemes as well as a variety of cryptographic accumulators. They can be widely applied in EFCB-IoT to meet not only integrity and confidentiality but some unique characteristics as well. We clarify which advanced primitives suit each part of the EFCB-IoT model explained in Fig. 2. Researchers can substitute them for the conventional primitives to suggest more solid and applicable privacy and security solutions.

## Proxy re-encryption scheme

***Outline***. Imagine that $\mathsf {Node}_1$ encrypted the message *m* to $C_{1}$ using its own key and stored it in a cloud or an intermediary fog. Clearly, no one could decrypt the encrypted data $C_{1}$, but the sharing of $C_{1}$ with other nodes would be pretty challenging. In IoT networks, nodes are willing to share their data stored on external servers with other entities. Proxy Re-Encryption (PRE) scheme is a relatively newfound encryption technique that securely shares the encrypted data stored on semi-honest or honest-but-curious clouds, fogs, and edges to other nodes.

*Solutions for secure sharing of encrypted data*. Assume $\mathsf {Node}_1$ and $\mathsf {Node}_2$ have their own private keys and shared their public keys with each other. There are three ways for $\mathsf {Node}_1$ to transfer the encrypted data $C_{1}$ which is stored on a cloud with $\mathsf {Node}_2$.

**Fig. 3:**
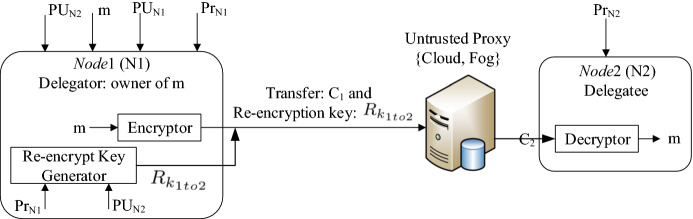
General diagram of a Proxy Re-Encryption (PRE) primitive

***a***) Decrypt-then-encrypt method: it is a trivial, slow, and costly approach. $\mathsf {Node}_1$ calls $C_{1}$ from the cloud, then decrypts it to extract *m* and finally encrypts again with $\mathsf {Node}_2$’s public key and sends to $\mathsf {Node}_2$.

***b***) Proxy-based method: the cloud as a trusted proxy owns both $\lbrace \mathsf {Node}_1$, $\mathsf {Node}_2 \rbrace$’s private keys. $C_{1}$ is decrypted to *m* and again encrypted to $C_{2}$ by $\mathsf {Node}_2$’s public key. Then, the proxy sends $C_{2}$ to $\mathsf {Node}_2$. This solution imposes less communication overhead, but all nodes have to trust the proxy.

***c***) Proxy Re-Encryption (PRE) method: PRE supersedes the two conventional solutions because it is substantially more efficient and brings supplementary properties of EFCB-IoT. The goal of PRE is the re-encryption of a ciphertext ($C_{1}$) encrypted by $\mathsf {Node}_1$ (delegator) to another ciphertext ($C_{2}$), which can only be decrypted by $\mathsf {Node}_2$ (delegatee).

The general structure of the PREs is depicted in Fig. 3$\mathsf {Node}_1$ has its private and public keys ($Pr_{N1}$ and $PU_{N1}$) and also the delegatee’s public key ($PU_{N2}$). $\mathsf {Node}_1$ is the unique entity that is able to extract the valid re-encryption key ($R_{k1to2}$). The proxy can re-encrypt the encrypted messages by $\mathsf {Node}_1$, like $C_{1}$. $\mathsf {Node}_2$ downloads the re-encrypted message ($C_{2}$) and decrypts with its private key ($Pr_{N2}$). Note that the proxy is not necessarily reliable and only owns the re-encryption key which is not enough to retrieve plaintext, unlike the former methods. and send to can be re-encrypted Having received the re-encryption key and ciphertext Neither $\mathsf {Node}_1$ nor $\mathsf {Node}_2$ trusts the proxy. $\mathsf {Node}_1$ generates the re-encryption key for the untrusted cloud/fog to extract $C_{2}$ from $C_{1}$. Obviously, the proxy cannot find any information about *m*.

***PRE benefits***. First, PRE considers IoT constraints if ciphertext transference is necessary. Although PRE schemes use the pairing transform functions, they substantially reduce the interaction and communication cost between clients and clouds, compared with the solutions; thus, the computation cost of nodes is thereby diminished. Also, the intrinsic characteristics of PRE considerably reduce the computation cost of IoT networks. Note that, as can be seen in Table 3, some of the PRE schemes are still computationally lightweight for very resource-constrained IoT platforms and each PRE supports some IoT-friendly functionalities. There are some blockchain-driven PREs align with the hierarchical structure of EFCB-IoT. A public blockchain can play the role of an untrusted proxy.

Also, the quantum-resistant ones resist the harvest-then-decrypt attack [109]. According to the striking development of quantum computers, we need PREs which can resist quantum attacks. Since the Shore algorithm [110] solves the number-theoretic problems in polynomial time, Hou et al. recently proposed not only quantum-resistant but also identity-based PRE over lattice sets [111]. Dutta et al. designed another quantum-resistant PRE which is collusion-resistant, non-transitive, and transparent [112]. In transparent PRE, the ciphertext $C_{1}$ encrypted by $\mathsf {Node}_1$’s key is indistinguishable from $C_{2}$ encrypted by the re-encryption key sent to $\mathsf {Node}_2$. In fact, the receiver nodes are not aware of the existence of a proxy in transparent PRE [113].

The noticeable advantages of PRE schemes such as key escrow free, decentralization, collusion resistance, and No SPOF show that PREs are TTPless-oriented, which is a valuable characteristic for deployment IoT in unattended environments. Furthermore, data owners can define various policies for sharing data and support access control mechanisms through PRE [114, 115].

***PRE Varieties***. We summarized different features of the most noticeable groups of PREs based on IBE and Attribute-Based encryption (ABE). Table 3 (page 18) compares PREs. Note that we only mention the recently published and IoT-friendly PREs with a few features to assist IoT. Each of them may be useful for a specific application, and we instantiate them in the next se case section.

**Table 3:** The comparison of IoT-friendly Proxy Re-Encryption primitives (SPoF: Single Point of Failure)

PRE schemes	Unique feature	Key Escrow free	Decentralized	Collusion resistant	Lightweight	Identity-based	No SPoF	Non-transitive	Bi-direction	Authorization	Revocation
Dent et al. [103]	Hybrid	–	–	$\checkmark$	$\checkmark$	–	–	$\checkmark$	–	–	–
Jiang et al. [104]	Encryption switching	–	-	–	–	$\checkmark$	–	–	–	–	–
Patil et al. [105]	Hierarchical structure	–	$\checkmark$	$\checkmark$	$\checkmark$	–	$\checkmark$	$\checkmark$	–	–	–
Su et al.[106]	Node revocation	–	–	–	–	$\checkmark$	–	–	–	$\checkmark$	$\checkmark$
Ahene et al. [107]	Signcryption-driven	$\checkmark$	–	–	–	–	–	-	–	$\checkmark$	–
Guo et al. [108]	Accountability	–	–	$\checkmark$	–	$\checkmark$	–	–	–	–	–
Hou et al. [111]	Quantum-resistant	-	–	–	–	–	–	$\checkmark$	$\checkmark$	–	–
Ahene et al. [116]	Non-repudiation	$\checkmark$	–	–	–	$\checkmark$	–	–	–	–	–
Koe et al. [117]	Offline delegator	–	–	–	–	–	–	–	–	–	–
Phuong et al. [118]	Puncturable encryption	$\checkmark$	–	–	–	$\checkmark$	$\checkmark$	–	–	–	–
Chunpeng et al. [119]	Broadcasting	–	–	$\checkmark$	-	$\checkmark$	–	–	–	–	$\checkmark$
Manzoor et al. [120]	Blockchain-based	$\checkmark$	$\checkmark$	$\checkmark$	$\checkmark$	–	$\checkmark$	–	–	–	–
Agyekum et al. [126]	Blockchain-based	–	$\checkmark$	–	–	$\checkmark$	–	$\checkmark$	–	$\checkmark$	–
Dutta et al. [112]	Quantum-resistant	–	–	$\checkmark$	–	$\checkmark$	–	$\checkmark$	–	–	–

- *Transitive/Non-transitive PRE*: A transitive PRE can send a ciphertext from $\mathsf {Node}_1$ to $\mathsf {Node}_2$ and then again from $\mathsf {Node}_2$ to $\mathsf {Node}_3$. A non-transitive PRE is merely allowed to share once. Thus, the ability of decryption can be re-delegated from $\mathsf {Node}_2$ to $\mathsf {Node}_3$ in transitive PREs which is a practical solution to connect different nodes in different clusters of Fig. 2.
- *One-directional/Bi-directional PRE*: if $\mathsf {Node}_1$ and $\mathsf { Node}_2$ mutually share their ciphertexts by proxy, it is bi-directional; otherwise, the PRE is one-directional. In a bi-directional PRE, both nodes, delegator and delegatee, have to generate and transfer re-encryption keys for the proxy.
- *Attribute-based PRE*: the attribute-based cryptographic primitives is a one-to-many encryption function that performs identity authentication at the same time. Every node has some attributes. If the attributes of the receiver (delegatee) match the attributes defined by the delegator, the ciphertext can be decrypted. There are two important groups of attribute-based PREs: ciphertext policy (CP-APRE) and key policy attribute-based encryption (KP-APRE). $\mathsf {Node}_1$ authorizes the proxy to convert $C_{1}$ according to access policy or a set of attributes in CP-APRE or KP-APRE, respectively. For instance, the location of nodes is a critical IoT-based attribute and can be considered before data is accessed. In Table 3, the schemes, which are not identity-based, are considered attribute-based.
- *Key Private (Anonymous) PRE*: The proxy, which performs the re-encryption phase, is unable to notice the identity of delegator, and delegatee because it deals with many nodes at the same time in anonymous PREs. Zhang et al. added the match-then-re-encrypt phase to PRE to formalize anonymous PRE [121].
- *Optimal/Non-optimal PRE*: In non-optimal PREs, each node has to protect all delegation keys and bears the striking expense of a Hardware Secure Module (HSM). In contrast, the users of optimal PREs only safeguard their private keys. The optimal PREs are useful for lightweight solutions in the first tier of EFCB-IoT architecture.
- *Non-interactive PRE*: If the re-encryption key is generated without $\mathsf {Node}_1$’s private key, the PRE is non-interactive. The interactive PREs are not appropriate for IoT platforms because of the high communication overhead.
- *Temporary PRE*: Proxy and $\mathsf {Node}_2$ can re-encrypt and decrypt, respectively only for a short period of time. In fact, $\mathsf {Node}_1$ can revoke the honored permission.
- *Collusion-resistant PRE*: If a malicious proxy colludes with a receiver (delegatee) to reveal the delegator’s private key, they do not succeed in collusion-resistant PREs. It is a vital feature in PREs because otherwise, a malicious proxy denies that it has been dishonest.

***PRE and IoT***. The notion of proxy is highly compatible with IoT paradigms. The following PRE-based use cases can consolidate IoT networks accompanied by more functionalities and security features.

${{\mathbf {\mathsf{{Use}}}}}$-${{\mathbf {\mathsf{{Case}}}}}$-${{\mathbf {\mathsf{{1}}}}}$ (*PRE and IoT interoperability*). PRE can work as a bridge between two deployed IoT systems with different assumptions. The delegator and delegatee in the majority of PRE schemes can interact with different types of encryption methods. There are some PREs called Hybrid PRE (HPRE) which convert from public PKI-based public key encryption to IBE and vice versa [104], or ABE to IBE) [103]. Therefore, this new utilization of PRE can strikingly improve interoperability between two formerly deployed IoT networks with different encryption schemes. Additionally, this service may be applied to encrypted data aggregation from different sources with other algorithms.

**Fig. 4:**
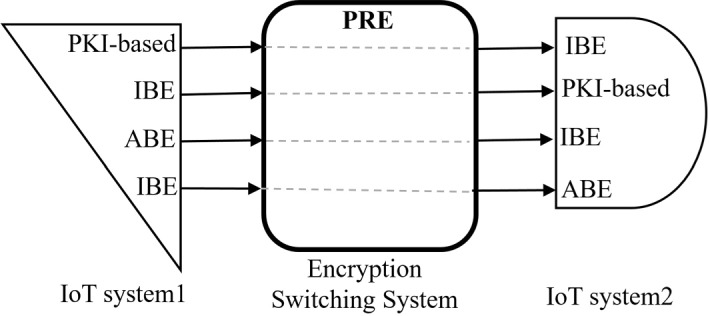
A schematic diagram of PRE as a proxy converter between different types of cryptographic primitives

Figure 4 is a conceptual model of designed hybrid PREs. Every cryptosystem in the rectangular can be converted into another cryptosystem in the semi-circle. It is a valuable advantage of PRE because it connects different IoT systems with distinct encryption methods. There is some research that each one partly establishes this practical switch. Deng et al. proposed a collusion-resistant and flexible HPRE to convert ABE-driven $C_{1}$ to IBE-driven $C_{2}$ [103]. Even if $\mathsf {Node}_2$ does not have the specified policies mentioned in an ABE (e.g., a specific name or location), it can access the IBE-driven ciphertext at a lower cost. Note that switching from IBE to ABE is still an open issue. The most noticeable weakness of this transformation is its complexity. They shoulder the burden of revocation and the addition of attributes and changing policies. Further, Jiang et al. designed a cross-domain encryption switching service based on a bi-directional PRE and bridged two well-studied encryption mechanisms, PKI-based public key encryption and IBE [104]. This scheme is much more efficient than the Deng algorithm [103].

${{\mathbf {\mathsf{{Use}}}}}$-${{\mathbf {\mathsf{{Case}}}}}$-${{\mathbf {\mathsf{{2}}}}}$ (*PRE and dishonest Edge/Fog/Cloud in IoT*). Most of the PRE schemes are designed based on a semi-honest or honest-but-curious proxy. Therefore, they are vulnerable to malicious behavior or key escrow drawbacks that cause the key abuse attack. If an authorized third party can access decryption keys from a covert channel or other circumstances, the encryption scheme is vulnerable to key escrow drawbacks. This problem is rooted in a high level of trust in trusted third parties [122]. Non-transferability, traceability, unforgeability of re-encryption key, authentication, and accountability are the different applied approaches to mitigate the key abuse attack by a malicious proxy [108].

Ahene et al. proposed a PRE that does not suffer from key escrow drawback risks and supports non-repudiation. They combined certificateless signcryption and PRE to design a pairing-free and integrity-driven PRE [107]. Further, the other signcryption-based PRE primitive proposed in [116] achieves non-repudiation, confidentiality, integrity, and authentication. This scheme is key-escrow-free, based on certificate cryptography, and has relatively fewer costs than other similar schemes. To prevent the malicious behavior of proxy, Guo et al. suggested an accountable PRE scheme. Imagine a proxy is accused to collude with some nodes and leak critical information of $\mathsf {Node}_1$, a judge can decide whether the proxy is guilty or innocent [108]. Their construction has public accountability and non-interactiveness but includes an extra judge algorithm.

Puncturable encryption (PE) is a forward secure encryption scheme for “store and forward” messaging. A forward secure encryption primitive periodically updates its secret key to keep the past encrypted messages confidential even if the key is compromised or misused. Although senders periodically update their decryption keys, the receivers do not require communication for the distribution of a new key [123]. Phuong et al. proposed a Puncturable PRE (PPRE) for asynchronous and many-to-many interactions such as group messaging services [118]. Since PE requires high computation overhead, using proxy as a puncturable encryptor is a pragmatic approach for lightweight devices. Their PPRE revokes the decryption capability only for some specific messages.

${{\mathbf {\mathsf{{Use}}}}}$-${{\mathbf {\mathsf{{Case}}}}}$-${{\mathbf {\mathsf{{3}}}}}$ (*PRE and decentralization in IoT*).

Although many PREs have been designed for centralized clouds. They require reliable nodes and have scalability problems, the decentralized cloud-based and blockchain-based ones can alleviate this issue.

Assume that a group of clouds, fogs, and edges desire to play the role of one proxy altogether. Patil and Purushothama recently expanded the idea of threshold PRE for this scenario. They designed a non-transitive, collusion-resistant, and threshold PRE for resource constrained networks, particularly for hierarchical IoT friendly networks [105]. The concept of threshold cryptography (secret sharing) is used to eliminate the central point of trust or semi-trust, and the distributed trust among a set of proxies. It also resists a single point of failure.

Some PREs have been designed for decentralized blockchain. Manzoor et al. proposed a blockchain-based and pairing free PRE scheme for secure IoT data sharing [120]. Guo et al. provided the first PRE to share encrypted data in a consensus algorithm of blockchain [124]. Chen et al. combined the concepts suggested in [105, 120], and proposed a threshold PRE based on blockchain [125]. Their main goal was the prevention of colluding between a single proxy and delegatee. Their scheme supports a group of proxies and needs a dealer, who selects and distributes the secret keys of all nodes. The dealer is an uncommon assumption for PREs and imposes a burden on the setup phase of the protocol. The re-encryption key is shared with *n* proxies by the delegator, and the *t* out of *n* proxies convert the ciphertext. Additionally, they proposed another scenario that the *t* proxies can reach a consensus on a consortium blockchain. Nodes can generate their keys by themselves without dealer participation [125]. Furthermore, Agyekum et al. recently designed an IoT-driven PRE based on blockchain. Their scheme used identity-based encryption to implement a simplified data-sharing platform [126]. Although the PRE doe not have additional properties, its performance is better than the other IBE PREs.

${{\mathbf {\mathsf{{Use}}}}}$-${{\mathbf {\mathsf{{Case}}}}}$-${{\mathbf {\mathsf{{4}}}}}$ (*Broadcast secure communication*). Broadcast PRE (BPRE) is another solution for sharing data with a group of receivers based in a cloud. Generating re-encryption keys for numerous delegates by a node is highly inefficient with the former discussed PREs. BPRE aims to reduce this computation overhead considerably. A proxy in a BPRE transforms a ciphertext of $\mathsf {Node}_1$ to a delegatee’s ciphertext, and no information about plaintexts is leaked to the proxy. Ge et al. designed a revocation-based BPRE [119]. Their scheme has a revocation list. As soon as $\mathsf {Node}_1$ adds a receiver in the revocation list, the proxy can re-generate the re-encryption key without knowing $\mathsf {Node}_1$’s private key.

Additionally, an offline PRE is proposed by Sandor et al. [117]. They address the always online demand of delegator for issuing re-encryption key and guarantee privacy through blind decryption. However, their scheme requires two TTPs as proxies. In addition, the nodes of IoT are particularly vulnerable to corruption. Consequently, the corrupted nodes must be revoked because they disrupt or fail the re-encryption process. Su et al. proposed the PRE scheme based on a trusted authorization on Cloud-IoT platforms to solve this problem [106]. Their PRE benefits from a permission process without affecting the other users.

## Wildcarded and downgradable encryption schemes

***Outline.*** In this section, we explain the varieties of Wildcarded Identity-based Encryption (WIE), and represent how they can be used in IoT networks. WIE is a kind of public-key encryption applied to selected multi-receiver settings. Abdalla et al. introduced the notion of wildcarded encryption in 2006 [127] and then proposed an identity-based one in 2011 [128]. The lately increasing research interest in the topic displays its importance and necessity. The sender of WIE can encrypt messages for a group of nodes with a particular pattern, a sequence of identities located in a domain. WIE is useful for downward communication from the cloud, fog, or even blockchain ledgers towards a group of nodes. In contrast to broadcast encryption and BPRE, only a distinct group of receivers can extract the plaintext, and the receivers may be variable for each transferred ciphertext.

**Fig. 5:**
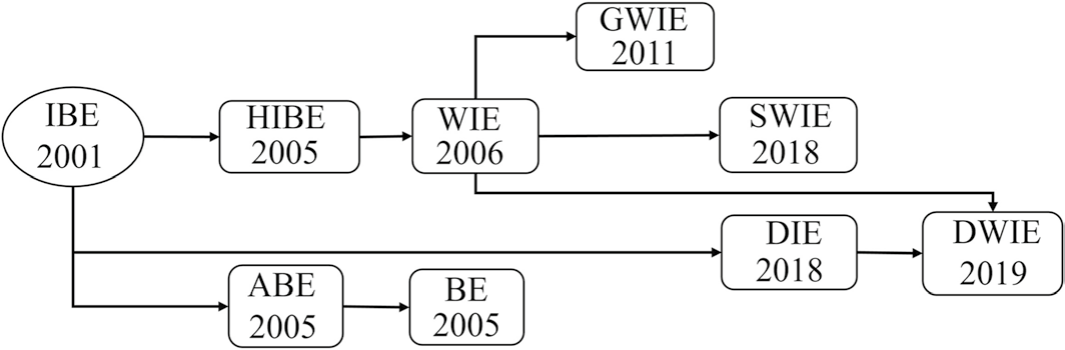
The evolutionary process of encryption schemes for one-to-many communication *IBE/IE* identity-based encryption, *HIE* hierarchical IE, *WIE* wildcarded IE, *GWIE* generalized wildcarded IE, *SWIE* Scalable wildcarded IE, *DIE* Downgradable IE, *ABE* attribute based-encryption, *BE* broadcast encryption

***WIE benefits***. They have two noticeable advantages for IoT networks. The first is that they are designed for multi-receiver settings in which an encryptor has then more autonomy to select a group of legitimate decryptors. A variable but precise group of nodes, as decryptors, should be able to retrieve the plaintext. Also, the computation cost is lower than other cryptosystems tailored to the one-to-many communication. We elaborate on the features with some examples in the following.

***WIE varieties***. Fig. 5 shows the progress on encryption primitives for multi-receiver settings from IBE to DWIE since 2001. In the following, we explain each part of the block diagram and mention the corresponding papers. First, we should clarify the difference between WIE, ABE, and broadcast encryption. Ciphertext-Policy-ABE (CP-ABE) has some commonalities with WIE and is currently being used in IoT systems. In 2020, Yu et al. used CP-ABE in IoT for the smart ocean to protect data privacy [129]. The difference between WIE and Ciphertext-Policy-ABE (CP-ABE) should be emphasized because they have the same functionality. It should be pointed out that the notion of WIE can be regarded as a simplified and restricted case of CP-ABE, but the computation cost of WIE is substantially lower than ABE. For example, Kim’s WIE scheme is 650 times faster than the constant-size CP-ABE [130]. Note that the notion of broadcast encryption which results from ABE has heavy computation overhead and places an intolerable burden on IoT nodes. Thus, WIE, which implies broadcast encryption, is a more efficient solution than the ABE-based cryptographic scheme.

Second, Abdalla et al. suggested an improved version called “Wicked-WIE” by allowing more general key delegation patterns [131, 132]. In Wicked-WIE, the wildcard symbol is used in nodes’ private keys, instead of a public key, to decrypt varied ciphertexts encrypted by several identities. However, the Wicked-WIE is less efficient than the WIE. Then, another scheme with generalized wildcarded key derivation (GWIE) was proposed in 2011. In GWIE, secret keys associated with pattern public keys consist of identities, and the wildcard symbol [133].

Apart from computation cost, the proposed WIEs had suffered from the large and increasing size of ciphertexts before Kim et al.’s suggestion. Recently, they proposed a Scalable Wildcarded Identity-Based Encryption (SWIE) appropriate for IoT systems because it generates a constant size of ciphertext regardless of the number of users [130]. Also, SWIE is 3 and 10 times faster than other existing WIEs mentioned in [127] and [131], respectively. Kim et al. extended their works and proposed a modified SWIE to achieve a higher provable security level. They provided practical pilot results based on IoT devices with 500 MHz Atom processor [135]. Duong et. al in [136] improved the Kim et al.’s scheme in [130]. Although both generate constant size ciphertext, Duong’s scheme has a shorter secret key size and less decryption computation time. The decryption process is almost 35 $\%$ faster. However, it is not scalable because it requires larger public storage than existing scalable ones.

Third, another related encryption scheme to WIE is Downgradable WIE (DWIE) as a new variant of identity-based encryption. Blazy et al. recently introduced (DIBE) and showed that DIBE can work with the conventional $\lbrace 0,1\rbrace$ alphabet, unlike WIEs with ternary alphabet $\lbrace 0,1,$ $* \rbrace$ [134]. For example, $\mathsf {Node}_1$ which owns the private key ($Pr_{ID}$) of $\mathsf {ID}$ can downgrade his key to another identity $\widehat{\mathsf {ID}}$ with this restriction that $\mathsf {Node}_1$ can only transform 1 into 0 in his identity string” [134]. If $\mathsf {Node}_2$ encrypts *m* with $\widehat{\mathsf {ID}}$ for $\mathsf {Node}_1$ and the downgraded $\mathsf {ID}$ matches $\widehat{\mathsf {ID}}$, $\mathsf {Node}_1$ can extract $Pr_{\widehat{\mathsf {ID}}}$ from ($Pr_{ID}$, $\widehat{\mathsf {ID}}$). Blazy et al. represent that any IBE with downgradable properties that can be transformed into DWIE. Therefore, there are two different WIE encryption schemes including IBE-oriented WIE and DIBE-oriented WIE. Although the SWIE is promising, avoiding wildcards makes DIBE more efficient than IBE.

Furthermore, Table 4 shows the difference between WIEs. The scalable ones manage to support newly arrived nodes. On the whole, SWIE is the fastest one. The identifiers of the group of receivers can be hidden in GWIE and SWIE. Both DWIE and SWIE reduce the ciphertext size to be constant regardless of the number of involved identities. WIE can be a secondary primitive in IoT devices for hierarchical cluster-based group messaging. Although ABE-based primitives can work in a multi-receiver setting, they impose extremely heavy computation overhead compared with WIEs. Thus, WIE primitives are much more IoT-friendly than the attribute-based encryption primitive.

**Table 4:** Comparison of WIEs (SWIE is the fastest one)

Scheme	Feature	Scalability	Pattern	Ciphertext size
WIE [128], 2011	Wildcarded	$\times$	Not-hidden	Variable
GWIE [133], 2012	Generalized	$\checkmark$	Hidden	Variable
SWIE [130], 2018	Scalable	$\checkmark$	Hidden	Constant
DWIE [134], 2019	Downgradable	$\times$	Not-hidden	Constant
SWIE [135], 2020	Scalable	$\checkmark$	Hidden	Constant
WIE [136], 2020	Wildcarded	$\times$	Hidden	Constant

***Wildcard and IoT***. As we mentioned before, the WIE family is beneficial for downward one-to-many multi-receiver downward communication, from the cloud to fogs, a fog to edges, and an edge device to a group of nodes in one cluster. The following use case clarifies how to apply WIE through an example.

${{\mathbf {\mathsf{{Use}}}}}$-${{\mathbf {\mathsf{{Case}}}}}$-${{\mathbf {\mathsf{{5}}}}}$ (*Selected One-to-many communication*). For example, imagine there is a cloud for a university called *SCIENCE* which is divided into two fogs (or cloud-lets) for different faculties, *MATH* and *LAW*. Each faculty defines different domains for a few departments $D_{1}$, $D_{2}$, $D_{3}$, $\ldots$, $D_{n}$, and every department has many staff (Nodes). We want to send an encrypted message for all nodes in *MATH*.$D_{1}$ including three users (*MATH*.$D_{1}$.$\mathsf {Node}_1$, *MATH*.$D_{1}$.$\mathsf {Node}_2$, and *MATH*.$D_{1}$. $\mathsf {Node}_3$). The conventional method is Hierarchical IBE (HIBE), but the message *m* has to be encrypted for each Node separately by HIBE. WIE is a more efficient method that can be applied in every hierarchical network. WIE encrypts *m* through identity with wildcard (*SCIENCE.MATH*.$D_{1}$.*) for all members of *MATH*.$D_{1}$ domain. Also, a node can send encrypted messages to every node in *LAW* faculty with Pattern=“*SCIENCE.* LAW.*.*” as the public key. The wildcard symbol (*) is added to identities to encrypt for a group of nodes simultaneously. The *pattern* is defined as a sequence of identifiers for a specific group of nodes. Patterns might be hidden or non-hidden in WIEs. A similar process can be performed in each part of the hierarchical EFCB architecture. Every entity in the tier 2 or 3 can use WIE to deliver an encrypted message to a selected entities in the lower layers.

## Registered-based encryption scheme

In this section, first, we discuss cryptographic accumulator functions as a prerequisite for registered-based encryption, and then we delve into the RBE schemes.

### Cryptographic accumulator

***Outline***. Generally, cryptographic accumulators (CAC) gather a set of parameters into a single root as a witness issued for commitment and membership proof. For example, the identities of *n* nodes, $X= \lbrace ID_{1},...,ID_{n} \rbrace$, are accumulated into the $Acc_{X}$. The issued $Acc_{X}$ is a proof of membership or witness for every participant node. If the security requirements of accumulators, including being one-way, indistinguishable, collision-resistance and undeniability are provided, the issued witness does not reveal any identity and supports anonymity [9].

***CAC benefits***. Although a CAC is not an encryption scheme and does not provide confidentiality per se, it is beneficial for IoT systems for two reasons. First, it is a significant prerequisite for the following section. Second, it is highly compatible with IoT networks because it provides integrity and immutability. Therefore, it assists in design of zero trust IoT-based security solutions.

**Fig. 6:**
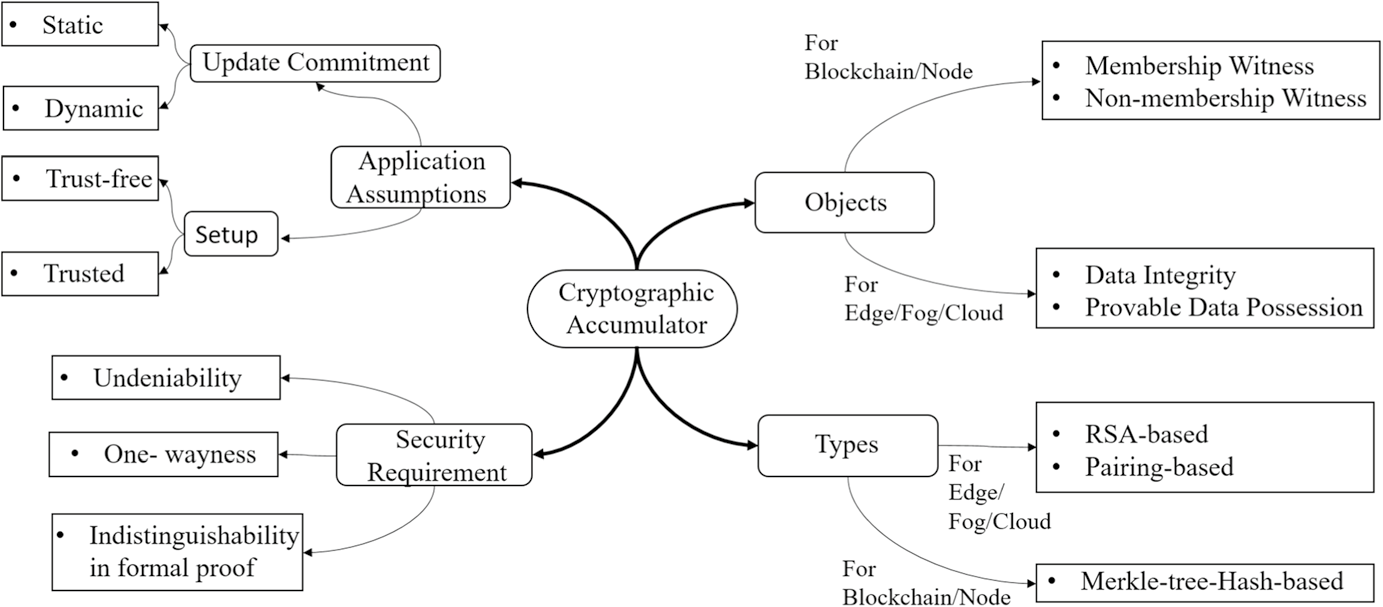
Untangling the different aspects of cryptographic accumulator

***CAC Varieties***. There are different types of cryptographic accumulators that suit diverse situations and back different characteristics. See Fig. 6 (page 25) to perceive the technical aspects of cryptographic accumulators. Each type can be suitable for the different parts of the IoT reference architecture, which is explained as follows.

- The conventional accumulators aid clients to own either a membership witness or a non-membership witness without revealing individual identity. The universal accumulators support both non-membership and membership witnesses. Also, some of them support undeniability and indistinguishability based on a unified formal model [139]. CACs with the objects can be merged with blockchain to manage the Nodes membership in the tier 1 of the EFCB-IoT.
- Accumulators have three major categories based on their building blocks, including hash-based, RSA-based, and pairing-based ones [9]. The hash-based accumulators are a variant of one-way hash functions based on the Merkle hash tree structure. The hash-based accumulators interestingly are trapdoor-less and drive without TTP. RSA-based and pairing-based accumulators work based on number-theoretic assumptions and require a TTP. The RSA-based ones satisfy one-wayness through the RSA hard problem. However, distributed RSA-based accumulators with batching can emulate a universal accumulator for a decentralized setting with no trusted entity [140]. According to the computation cost of RSA-based and pairing-based CACs, they suit higher entities like edge, fog, and cloud. The Merkle-tree-based ones are appropriate for lower devices.
- There are two different categories of accumulators including dynamic and static. Dynamic accumulators can efficiently update commitments and membership proofs that stem from added or removed elements from the set. However, static accumulators lack commitment updating. Both static and dynamic ones can be constructed based on the mentioned RSA, bilinear pairing, and Merkle hash tree types [140].

***CAC and IoT***. Accumulators contribute to IoT devices in two ways. First, nodes can prove their membership in a specific IoT system. Second, they can be used as a building block of other primitives such as in time-stamping techniques, anonymous credentials, registration-based cryptography, ring signature, and the decentralized structure of blockchain. The Merkle tree structure is a simple accumulator [137]; however, more features have been introduced in more recently proposed accumulators for different best practices. For example, new features were applied to Zerocoin shaping it as the most anonymity-supported cryptocurrency [138]. Due to the conformity between cryptographic accumulators and hierarchical IoT structures as well as blockchain technology, we will progressively see more accumulator-oriented security solutions in IoT platforms.

${{\mathbf {\mathsf{{Use}}}}}$-${{\mathbf {\mathsf{{Case}}}}}$-${{\mathbf {\mathsf{{6}}}}}$ (*Lightweight Blockchain*). Boneh et al. recently designed an accumulator based on batching and aggregation techniques for TTPless settings. It provides the same functionality as accumulators for an ordered list of elements in public blockchains, where nodes only need a constant amount of storage in order to participate in a heavy consensus algorithm. Their scheme minimizes the growth of network communication. Replacing conventional Merkle trees with the vector commitment accumulator reduces roughly 80% verification time [140].

${{\mathbf {\mathsf{{Use}}}}}$-${{\mathbf {\mathsf{{Case}}}}}$-${{\mathbf {\mathsf{{7}}}}}$ (*CAC on cloud and fog*). Accumulators check the integrity and possession of sensitive data stored in cloud/fog storage through the owners of data. Also, they can detect any unauthorized manipulation of uploaded data in the cloud even with the owner of the cloud. It should be pointed out that there are some probabilistic methods designed for cloud integrity verification. These methods randomly check some chosen data blocks. Accumulators conduct a deterministic, provable, and private verification of integrity and provide a full guarantee that all data frames are correct and intact [141]. Khedr et al. recently proposed an efficient RSA-based accumulator called BlockGen that is secure against any forgery, data deletion, replacement, and data leakage. Meanwhile, it supports the delegation of responsibility for integrity verification to another auditor. Computation and communication costs of their scheme are negligible compared with similar methods [142].

### Registered-based encryption

***RBE outline***. IoT entities, ranging from small gadgets to high-end servers and blockchains, use variants of public-key cryptography. PKI-based and IBE-based primitives are the two major and conventional categories of public-key cryptography with distinct benefits. In this section, we discuss Registered-Based Encryption (RBE) as a recent category of PKC proposed in 2018 that covers some benefits of both types and working without TTP.

***RBE benefits***. RBE fundamentally tackles a major functional problem in all cryptosystems. It does not require any TTP for the setup phase in the beginning. On the one hand, PKI-based systems need at least a TTP to extract the public key of nodes from the private key. Not only is the public key string long and meaningless, but digital certificates also have to be applied for binding the public key and identity. Furthermore, adversaries might apply for a few distinct public keys with different identities and use them for malicious activities [143]. On the other hand, Shamir proposed the idea of Identity-based encryption in 1984 [144]. After 17 years, Boneh and Franklin introduced the first IBE encryption scheme [145]. Over the last few decades, various IBE primitives have been proposed. IBE reduces the burden of key distribution overhead; however, they all still require a TTP as a PKG to generate public/private keys by its master key.

Having TTP in encryption schemes might cause some drawbacks. For example, key escrow is the first issue in which a PKG might arbitrarily decrypt nodes’ ciphertexts without permission and violate their security and privacy. Second, a TTP may be inconsistent with the IoT platform because the lack of trust is an attribute of IoT systems deployed in unattended areas or which are connected with public blockchains. Therefore, TTP is not always available in IoT. Moreover, if a TTP faces a breach (e.g., its master key is compromised), the security of the entire system may be violated. It means that the TTP as a SPoF is the enemy of availability in IoT systems.

RBE is a recently proposed identity-based encryption scheme which has no TTP entity and entirely rectifies the key escrow issue. Although other solutions, including de-centralized multi PKG, PKG accountability, and certificateless PKC, have been successful to diminish the side effects of key escrow, RBE eradicates such a problematic issue.

**Table 5:** Comparison of registration-based encryption primitives

Scheme	TTP-Less	Accumulator	Public Verifiability	Best practice	Extra
RBE [146]-2018	$\checkmark$	Honest	$\times$	–	–Not efficient
RBE [148]-219	$\checkmark$	Honest	$\times$	Cloud service	+Anonymity
VRBE [147]-2020	$\checkmark$	Malicious	$\checkmark$	Blockchain service	+Slightly efficient
ORBE [149]-2021	$\checkmark$	Honest	$\times$	–	+Efficient

***RBE Varieties***. The varieties of RBE Garg et al. proposed the first version of RBE in 2018 which is weakly efficient under standard assumptions [146]. Then, they proposed a noticeably faster and improved version in [148]. The latter RBE can be extended to an anonymous RBE. The well-designed RBE in [148] is more efficient than the first one in [146]. The authors used the red-black Merkle tree and timestamp-RBE subroutine to become efficient. However, both schemes imagined that the accumulator is an honest-but-curious entity.

However, a malicious or corrupted key accumulator can potentially fail the RBE by using secretly registered multiple keys for already registered users or register any key for currently unregistered users. There are two solutions to ease such a problematic issue. The first fairly inefficient approach applied in [146, 148] is public auditability via rebuilding public parameters and comparing it with the accumulated public parameters. The second approach is public verifiability proposed in [147].

Recently, Goyal and Vusirikala modified RBE to resist a malicious key accumulator [147]. They proposed Verifiable RBE (VRBE), in which users can obtain short proofs from the key accumulator proving correct registration. It provides the proof of correct registration for registered users as well as the proof of non-registration for unregistered entities [147]. The proof system is more efficient than public auditability in [148]. Also, the size of ciphertexts in VRBE is smaller than RBE. VRBE has two more pre/post-registration proofs to ensure that the key accumulator behaves honestly. This process is done on a randomly chosen small subset of users to prevent accumulator misuse. The very large ciphertext size is a major issue in the discussed RBEs. Cong et al. optimized the first RBE [146] and designed an RBE with 57.5% smaller ciphertext and 30% less computation cost of decryption [149]. They replaced Merkle tree with crit-bit trees. Table 5 highlights the key features and differences of all proposed RBE primitives. RBE is a sophisticated and promising primitive for IoT systems.

***RBE and IoT***. The unique characteristics of RBE make it possible to design a TTP-less IoT structure, which is demanding for unattended areas.

**Fig. 7:**
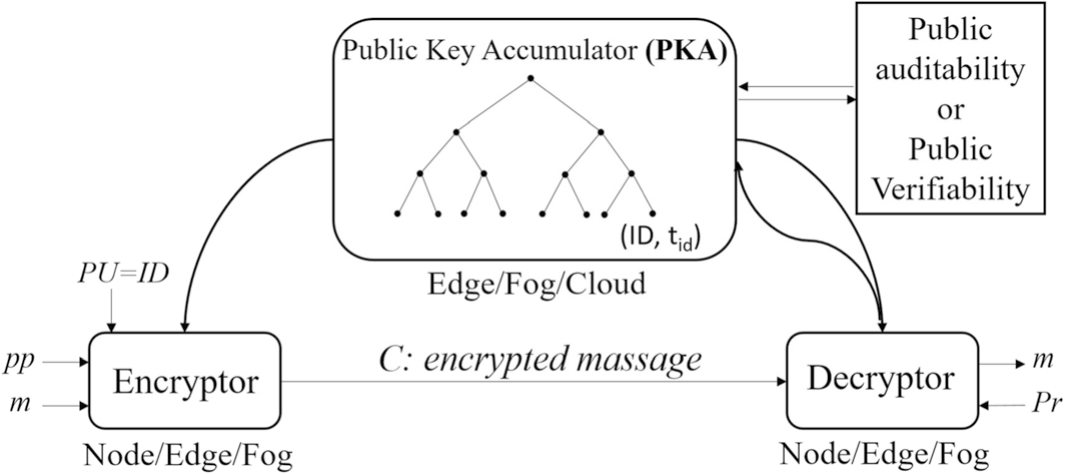
The high level structure of registration-based encryption

${{\mathbf {\mathsf{{Use}}}}}$-${{\mathbf {\mathsf{{Case}}}}}$-${{\mathbf {\mathsf{{8}}}}}$ (*Zero Trust IoT platform*). Figure 7 shows the high-level depiction of RBE in EFCB-IoT architecture. Each layer can play the role of a PKA for the lower layer, which means that two nodes, two edges, or two fogs can exclusively transfer encrypted messages. Blockchain can also aid in keeping the PKA more accountable and satisfy public verifiability. We explain how Fig. 7 works as follows.

In Fig. 7, RBE is aligned with the hierarchical structure if EFCB-IoT. The lower entities can have confidentiality even if they distrust the higher entities. Assume RBE has three entities: encryptor, decryptor, and Public Key Accumulator (PKA). The encryptor can be a node/edge/fog, and the decryptor can be edge/fog/cloud respectively. Every node generates its pairwise private-public keys ($PU=ID$, *Pr*), and registers the public key in the PKA, which has no secret key. PKA adds *ID* to the list of registered identities into a Merkle-tree structure with a time-stamp ($t_{id}$) for fast binary search. The PKA only compresses identity-key pairs and publishes the updated tree as public parameters (*pp*). PKA includes the public key of all registered nodes with their $t_{id}$, and this means that PKA is a reference monitor to connect the encryptor and decryptor. It is fully auditable and has no secret key [146]. The encryptor takes as input the *ID*, message *m*, *pp*, and $t_{id}$. Then, it outputs a ciphertext *c*, which is obtained by using the time-stamp corresponding to *ID* ($t_{id}$). Thus, the encryptor firstly requires to lookup *ID* in the tree structure. Note that all users have to receive the fresh public parameters *pp* for encryption [147]. Then, any honestly registered user can decrypt *c* with *Pr*. The RBE decryptor interacts with the timestamp-RBE function to obtain supplementary key parameters.

RBE primitives should cover three pillars to be efficient. First, public parameters have to be short enough. Second, the registration process has to be highly efficient, and also the updating of public parameters received from PKA has to be done in polynomial time. Moreover, there are two methods to interact with PKA: time-restricted and time-unrestricted. The former gives nodes a short period for registration, but in the latter, users are allowed to register at arbitrary time intervals.

## Break-glass encryption

***Outline***. In some scenarios such as healthcare systems, data criticality outweighs data confidentiality and vital data must be immediately available. “Break-glass” is an idiomatic term used to explain the emergency access to encrypted data in the cloud. Although some emergency break-glass *access control* mechanisms have been designed to cope with this situation in IoT networks [150, 151], Scafuro formally defined the notion of Break-Glass Encryption (BGE) in 2019 [152]. BGE means that the encrypted messages on cloud storage can be violated *just once* for *an emergency*, and *without* the primary decryption key by the honest-but-curious or untrusted cloud/fog storage. The most challenging part of BGE is whether a node is legitimate to break the glass or not. This vital access is *publicly detectable* without relying on a TTP. BGE is a very captivating and sophisticated *private-key* primitive for IoT systems tailored to critical infrastructure. Numerous nodes store their encrypted data on an *untrusted* cloud/fog entity or private blockchain.

***BGE benefits***. Apart from confidentiality, detectability and accountability are of utmost importance. Detectability makes remote storage accountable. The illegitimate break-glass procedure should be detectable. Furthermore, BGE-based access controls are closely aligned with EFCB-IoT architecture, especially for healthcare and cyber-physical systems.

There are two misuse scenarios that should be prevented in a secure BGE scheme. First, an honest-but-curious server might break all ciphertexts in an apparently critical situation. If it violates a ciphertext without any permission, it will be traced owing to the detectability of BGE. Second, a malicious $\mathsf {Node}_1$ might request for breaking the ciphertext of $\mathsf {Node}_2$. The cloud would send an alert to $\mathsf {Node}_2$, and then delegitimize $\mathsf {Node}_1$’s request if $\mathsf {Node}_2$ answers in a certain interval of time because it means that $\mathsf {Node}_2$ still possesses its secret keys. Therefore neither server nor another node can violate security and perform one of the attacks.

Note that there is no secure alternative primitive to the break-glass functionality. Imagine, $\mathsf {Node}_1$ that has uploaded its encrypted data on the cloud gives its secret key to another allegedly reliable node or a group of nodes through secret sharing for use in critical condition. They can collude with the cloud entity because key transferring lacks detectability that violates accountability. Thus, BGE owns unique characteristics with no alternative primitive.

Furthermore, we should clarify the difference between the notion of break-glass and key-escrow. In key-escrow-based encryption schemes (e.g., commercial RSA and IBE), a TTP can undetectably decrypt all messages many times. However, an emergency decryption in BGE is detectable and can only be performed one time. All storage (cloud/fog/edge) is kept under surveillance of all nodes by BGE.

***BGE varieties***. The scheme proposed in [152] needs stateful trusted hardware, which requires global clock synchronization while preserving semantic security for cloud and blockchain settings. Yang et al. proposed a lightweight password-based break-glass system healthcare IoT that supports two ways of accessing encrypted data [153]. Their system is built based on the pairing transform. Padmashree et al. used elliptic curve cryptography, instead of the pairing-based, and reduced the size of ciphertext and time complexity [154].

***BGE and IoT***. Today, many vendors provide IoT services for its ramification such as big data analysis. BGE can be a beneficial primitive for privacy preservation through the detection of violations for working in this IoT ecosystem. Furthermore, BGE is essential for IoT-oriented infrastructures in healthcare or cyber-physical systems. Although the concept of break-glass encryption is an original idea, it is rather impractical and needs more feasibility studies and improvement. A formal definition of BGE is provided in [152]. Bael et al. recently instantiated an emergency break-glass scheme for IoT environments [156]. We will probably hear much more of BGE before long.

A BGE has three functions:* Encryption*, *Decryption*, and *Break*. The cloud and a legitimate user cooperate to perform *Break* and gain access to sensitive data. Honest-but-curious cloud/fog services are the best spots for BGE deployment. BGE can keep the reputed vendors (e.g., Google Drive, Azure, IBM blockchain, iCloud, Cisco, Hivecell) reliable because they avoid loss of reputation against unpermitted and detectable decryption of ciphertexts. Thus, BGE should not be applied appropriately on an unknown cloud which is not accountable. Also, BGE based on permissioned blockchain technology can address this issue. Scafuro suggests a BGE implementation using a blockchain [152].

${{\mathbf {\mathsf{{Use}}}}}$-${{\mathbf {\mathsf{{Case}}}}}$-${{\mathbf {\mathsf{{9}}}}}$ (*Disaster Recovery*) It actually can be considered as three different use cases. Suppose one of the three following conditions is satisfied. Then, the intermediary entities break the glass, securely retrieve the encrypted data and reveal the original message.

**Table:** 

*IF * $\lbrace$	
1) Nodes lost their keys that they have used for stored ciphertexts encryption.	
*OR*	
2) The encryptor node as the only owner of a primary key was destructed and cannot extract the corresponding plaintexts anymore.	
*OR*	
3) There was an emergency condition and access to the key for decryption was time-consuming.	
(e.g.,it is likely in healthcare systems or critical infrastructures)	
$$\rbrace$$	
*THEN* $\lbrace$	
The intermediary storage (Cloud/Fog/Edge) without the primary secret key reveals the plain message only once for a legitimate or a representative user.	
$$\rbrace$$	

${{\mathbf {\mathsf{{Use}}}}}$-${{\mathbf {\mathsf{{Case}}}}}$-${{\mathbf {\mathsf{{10}}}}}$ (*Modified Bell Lapadula Access Control*). The Bell-LaPadula model is conventional confidentiality-driven access control for the information flow in a multi-level structure. It has two strict rules, *no read up* (single property) and *no write down* (star property). The former states that an entity cannot read the information at a higher level, and the latter states that an entity cannot write information at a lower sensitivity level [155]. Although this model strongly mitigates the confidentiality risks, it hinders availability, particularly in contingency and disaster recovery plans. BGE improves the Bell-Lapadula expressing policies and facilitates access without security compromising [157]. The BGE-based Bell Lapadula approach keeps availability in some specific scenarios by breaking the *no read up* and implementing the * read up* rule. Some lower subjects can access a few higher classified contexts.

## Integration of primitives

**Fig. 8:**
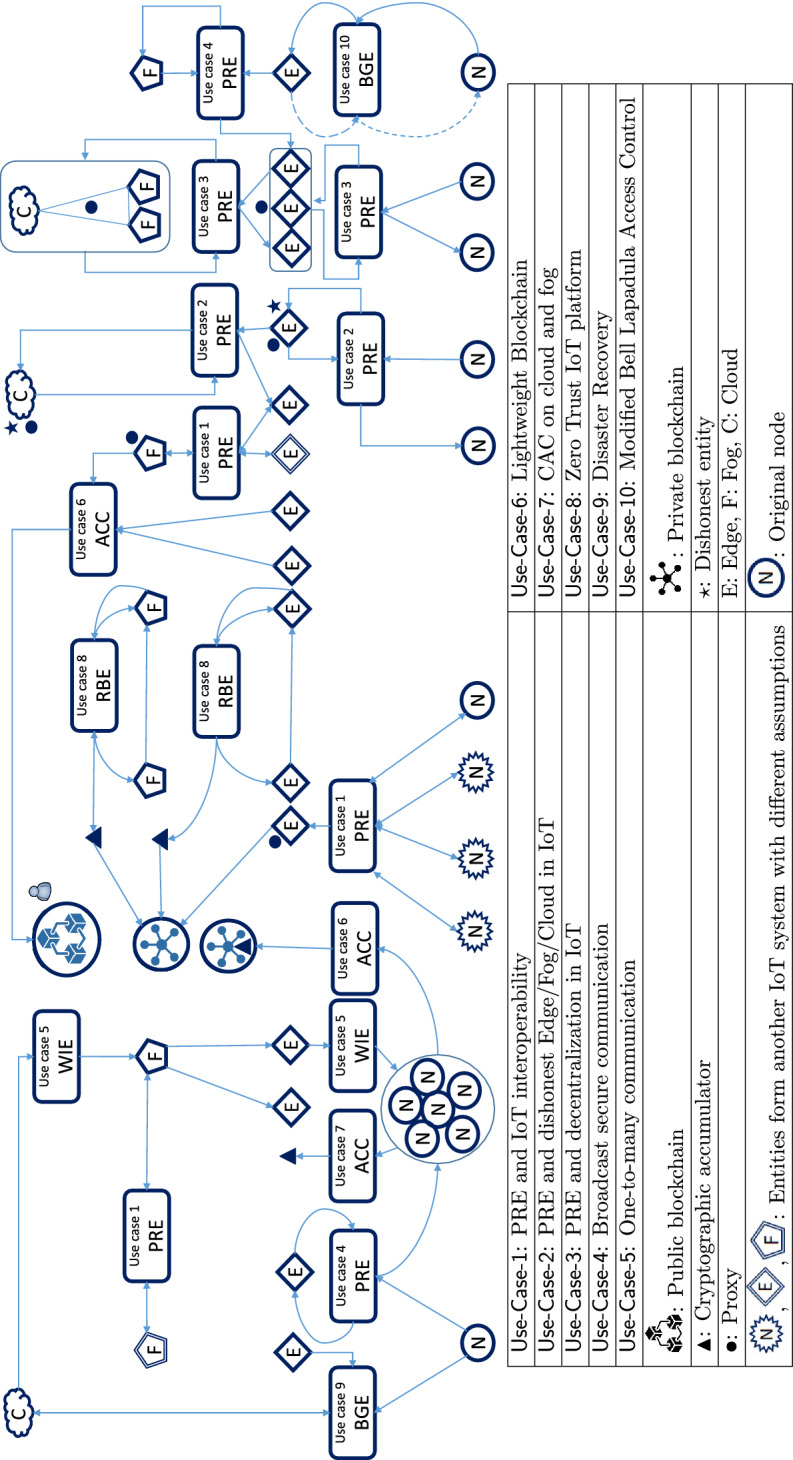
The conceptual model of the advanced encryption schemes’ use cases in EFCB IoT architecture (*PRE* proxy re-encryption, *WIE* wildcarded identity-based encryption, *RBE* registration-based encryption, *BGE* break-glass encryption, *CAC* cryptographic accumulator)

This section summarizes all discussed cryptographic primitives and elaborates on how the discussed cryptosystems and use cases can work together based on the EFCB-IoT architecture through the conceptual diagram. Then we elaborate how to achieve a specific characteristic through the advanced encryption schemes. Finally, we depict how use cases can be combined, emphasizing the usability and relevancy of the advanced cryptosystems in IoT networks.

***A***-**Conceptual model**. Figure 8 represents the suggested spots of the discussed use cases in the EFCB-IoT model. We explained how the current problems in the introduction section, including placing less trust in IoT entities and taking advantage of various additional functionalities, can be addressed. The location of every use case is an instantiation, and it can be used in other parts of the network when we are looking for the same characteristics.

- In $\mathsf {Use}$-$\mathsf {Case}$-$\mathsf {1}$, the entities from other IoT systems with different cryptographic assumptions can interoperate through a PRE. Note that the mentioned proxies are pointed by “$\varvec{\bullet }$”. This use case is spotted three times in separate tiers. Two fogs in same tier but with different assumptions can have a horizontal connection. Without shared keys for a mutual connection, Some node or edge devices can have vertical communication with their corresponding higher tier. Each proxy can bridge the gap and translate the mutual communication with two different assumptions. The proxy can send a summary to a local blockchain as requested. The local private blockchain can be used to monitor the proxy activities for preventing any repudiation. Furthermore, since the nodes are resource-constrained, the *E* can play the role of proxy server and manages burdening storage and computation overheads.
- $\mathsf {Use}$-$\mathsf {Case}$-$\mathsf {2}$ are mentioned twice in the conceptual model. A dishonest cloud or edge can mediate for vertically sending messages from the lowest tier to the highest tier. The lower IoT entities of the $\mathsf {Use}$-$\mathsf {Case}$-$\mathsf {2}$ (N and E) can monitor the dishonest and malicious higher entities (E and C), respectively.
- Also, a group of edge devices or a group of fog and cloud entities can play the role of a proxy in the $\mathsf {Use}$-$\mathsf {Case}$-$\mathsf {3}$. It helps nodes to place less trust on the intermediary proxies, which increases the network reliability. Also, a public blockchain can assist a proxy to keep the history of all delegations including the identity of the delegator and the delegatee.
- $\mathsf {Use}$-$\mathsf {Case}$-$\mathsf {4}$ provides one-to-many communication among same-level entities. An edge sends some data to the higher, and then it as a proxy sends it to a group of edge devices. Similarly, a group of nodes have secure mutual connections with the in charge edge entity. The group also keeps interaction with distant nodes.
- $\mathsf {Use}$-$\mathsf {Case}$-$\mathsf {5}$ provides hierarchical one-to-many communication with selective destinations is delivered. As can be seen, a cloud establishes downward communication with selected fogs and edges. Similarly, an edge can launch one-way connection with selected nodes in tier 1.
- $\mathsf {Use}$-$\mathsf {Case}$-$\mathsf {6}$ is recommended twice in different layers. The synergy between CAC and blockchain is realized by this use case. The close cooperation between blockchain and CAC-based PKA can set up encryption without any TTP.
- $\mathsf {Use}$-$\mathsf {Case}$-$\mathsf {7}$ calculates a digest of transcripts in a local cluster through a supplementary accumulator. The tier 2 requires an storage to check the integrity and ownership before aggregation or sending to the upper layers.
- $\mathsf {Use}$-$\mathsf {Case}$-$\mathsf {8}$ that is depicted twice in the center of Figure 8 for direct (no proxy) connection without any TTP. The local blockchain can be regarded as the public key accumulator to provide public verifiability.
- Also, The E has access to the encrypted data generated by the node on the cloud in an emergency in $\mathsf {Use}$-$\mathsf {Case}$-$\mathsf {9}$. If the network loses some nodes, which is not unlikely, this use case is fully functional.
- The dashed line in $\mathsf {Use}$-$\mathsf {Case}$-$\mathsf {10}$ is the additional access provided by BGE. As we discussed earlier, it makes IoT systems more adjustable when using strict confidentiality-driven access control, like Bell-LaPadula.

**Table 6:**
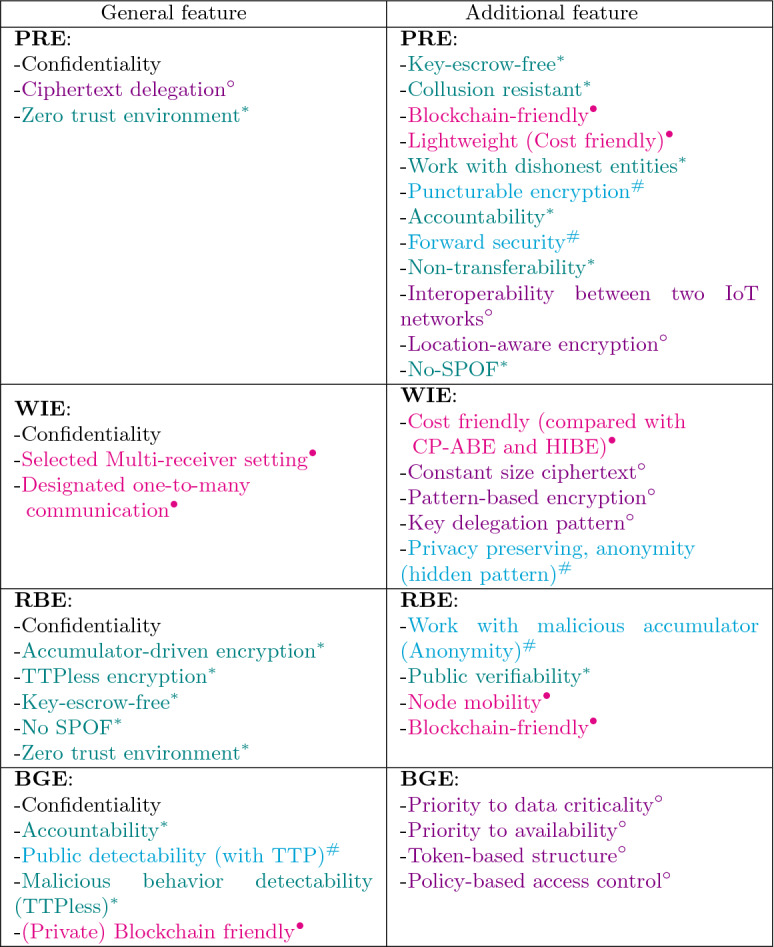
Comparison of the grouped characteristics of the advanced cryptosystems

(Grean$^{*}$: aligned with zero trust environment,

Pink$^{\bullet }$: Compatible with EFCB-IoT structure,

Violet$^{\circ }$: Supplementary functionality,

Cyan$^{\#}$:Privacy-preserving property)

***B***-***Connecting the dots***. Table 6 summarizes all characteristics of the discussed cryptosystems and represents the relationship, commonalities, and discrepancies with different colours. This big picture assists in securing various IoT networks with different assumptions. The general features (first column) are standard among all schemes of each cryptosystem. The additional features (second column) are provided by a few specific schemes that we discussed in the former sections.

Although we cited and compared the corresponding papers in the preceding sections, we recap the mentioned primitives altogether to provide the following specific features, that are clarified as challenges in IoT systems in the Introduction section.

1. *Privacy-Preserving*: Privacy has different aspects that each one might be prioritized according to various practices. Among the surveyed encryption schemes, two family of algorithms provide two privacy features. The WIEs with hidden patterns [130, 133] provide unlinkability, and the anonymous RBE in [148] provides anonymity.
2. *TTP-less Structures*: For unattended or unreliable environments such as a battlefield or jungle, using TTP-less solutions is highly recommended. However, rarely do encryption schemes manage this situation in practice. As we discussed, RBEs require no TTP [146–148] and are valuable assets for massive IoT networks
3. *Misbehavior prevention and detection*: Apart from RBE, the deployment of many cryptographic primitives, particularly the setup phase, without a TTP is inevitable. Some IoT-based applications count on TTP or PKG support for key management and supervision. Thus, some preventive measures for controlling trusted entities can mitigate their malicious activities. We discussed a set of primitives with this significant attribute recapped as follows:
  The PRE primitive [105, 108, 119, 120] is collusion-resistant.
  The BGE [152] fitted for mission-critical networks inherits misbehavior detection.
  Even with a malicious accumulator, the RBE [147] works properly owing to public verifiability.
  The key escrow issue can violate privacy and security. The key-escrow-free PREs designed in [107, 116, 118, 120] can diminish the dominance of the centralized entities including fogs and clouds in IoT architecture.
4. *Defense in Depth* is an approach to use a series of security mechanisms to cautiously protect data. This approach is generally considered network architecture; however, the PREs proposed in [105, 118, 120] resist SPoF and therefore work even with a failed server.
5. *Blockchain Friendly* means the corresponding cryptosystems’ use cases drive blockchain as an entity. Although all encryption and digital signature schemes, ranging from conventional to advanced ones, can potentially work with blockchain technology, the ones which intrinsically support a hierarchical structure conform better with blockchain. Cryptographic accumulators [140, 142] provide integrity. Also, RBE primitives are blockchain-friendly [146, 148].
6. *Quantum resistance*: Though post-quantum cryptography was out of the scope of this paper, the bi-directional PRE in [111] is a quantum-resistant primitive among the discussed advanced primitives. Caramés in [20], Lohachab et al. in [16], and Caramés & Tiago in [158] elaborated quantum-resistant encryption algorithms in IoT systems.

***C***-***Combination***. We surveyed the advanced cryptosystems, designed many use cases, and localized them on the EFCB IoT architecture. Then we showed them how their characteristics are related and how to alleviate the IoT challenges in four groups of features. In some scenarios, we may require some of them alongside each other to achieve some goals in one action. Thus we discuss their combination. We are mentioning some synergistic IoT-driven combinations of the mentioned cryptosystems. We apply them together to attain the both-sides features of two cryptosystems.

1. *Multi-receiver proxy re-encryption* ($\mathsf {Use}$ $\mathsf {Case}$ $\mathsf {1}$+$\mathsf {5}$). We might have multiple receivers in different IoT systems with inconsistent cryptographic assumptions. In this scenario, the combination of PRE and WIE can drive upward and downward communication among entities and realize wildcarded interoperability. It is a demanding application for connecting the IoT systems that have been established based on different standards.
2. *Wildcarded broadcast proxy re-encryption* ($\mathsf {Use}$ $\mathsf {Case}$ $\mathsf {4}$+$\mathsf {5}$). It is a pragmatic approach for sending an encrypted message for only some selected destinations in a group of edge devices or nodes. A cloud takes the responsibility of both a proxy and a selector to define the specific pattern as a sequence of identifiers of receivers.
3. *Proxy decentralized wildcarded encryption* ($\mathsf {Use}$ $\mathsf {Case}$ $\mathsf {3}$+$\mathsf {5}$). Similar to the former one, a blockchain or a group of high-level entities in the EFCB architecture is a proxy to convert some ciphertexts to only a few specified nodes. We may require some secret-sharing or consensus algorithms to meet decentralization among the IoT entities.
4. * Registration-based wildcarded encryption* ($\mathsf {Use}$ $\mathsf {Case}$ $\mathsf {5}$+$\mathsf {8}$). This combination is a substantial improvement. WIE schemes require a TTP in the initialization phase and RBE aims to work without a TTP. Thus, combining RBE and WIE discards the requirement, makes WIE available for zero-trust environments and takes advantage of various supplementary functionalities of WIE. For example, a PKA and a blockchain may be applied instead of the superior cloud.
5. * Decentralized break-glass proxy re-encryption* ($\mathsf {Use}$ $\mathsf {Case}$ $\mathsf {3}$+$\mathsf {9}$). This association between PRE and BGE relies on less trust because a group of entities plays the role of the *Breaker* who have got permitted by the data owner. Each entity is accountable, but they could not break the glass and obtain personal information without collaboration. Therefore, it is a defense-in-depth strategy and reduces privacy risks.

***D***- ***Future research directions***. Although this paper surveyed the advanced encryption schemes which mainly involved future literacy, they are not security elixir for IoT systems, and there is room for improvement that will stem from greater innovative step-forward research. n the following, we mention four separate roadmaps about the required cryptosystems that will be heard more in the future. The last one includes four separate sub-items.

1) As we mentioned, the EFCB-IoT model is a promising combination of centralization and decentralization. It is very functional, but we should finally embrace pervasive and completely decentralized computing and networks for some applications. Therefore, all security mechanisms, including cryptosystems, should be reformed based on egalitarian assumptions. Although this paper’s discussed encryption systems and the designed use-cases can somehow meet decentralization assumptions, manage trust, and operate without trusted parties, this research orientation will be increasingly demanding.

2) The distributed ledger technology in blockchain contributes to decentralization by transparency and immutability. Still, it might undermine some privacy aspects and may cause some highly questionable challenges in privacy protection [159]. Privacy is an umbrella term of different terminologies, such as unlinkability, untraceability, anonymity, and forward security. Also, General Data Protection Regulation (GDPR) upholds the principles of data minimization, including the right to correct data by the owner, the right to be forgotten, and the right to restrict processing [160]. Thus some intrinsic issues in blockchain structure violate privacy, and we should consider privacy by default at the beginning of building every blockchain-driven product. From a cryptography point of view, the most prevalent cryptosystems are not also thoroughly GDPR-compliant. The standard cryptosystems might hinder the adoption of privacy-preserving decentralized technologies. Furthermore, GDPR is an instantiation of privacy rules, and we will hear more about related regulations and market demands. For example, the data flow inside of the Artificial intelligence (AI) model should be hidden in 6g-IoT networks [161] and the integration of federated learning into EFCB-IoT is challenging without privacy-preserving encryption methods in blockchain systems [162]. Therefore, privacy-enhanced decentralized encryption mechanisms are another encouraging research roadmap. However, other cryptographic techniques, such as zero-knowledge proof and digital signature, have the potential to improve privacy.

3) Future networks should resist quantum computing threats by using quantum-resistant encryption algorithms. Large-scale quantum computers will be built in the future and break the conventional cryptosystems. Thus, we should proactively design advanced post-quantum cryptosystems before beginning the pervasive post-quantum era. Although we discussed a few quantum-resistant PREs, we require considerably more quantum-safe advanced cryptosystems with more functionalities and less trusted parties as a prerequisite. Therefore, quantum-resistant PRE, WIE, RBE, and BGE will be demanding shortly. Note that some homomorphic encryption methods are quantum-resistant [163]. Advanced encryption schemes driven by homomorphic solutions will be a promising approach to achieving two features by performing one algorithm. In addition, quantum cryptography is another promising approach that is based on quantum mechanical phenomena. Quantum key distribution is another practical solution that works in combination with symmetric encryption [164].

4) In the following, we discuss some new ideas and road maps for further research specifically related to the four encryption schemes discussed in this paper.

- We mentioned a variety of use-cases for each cryptosystem. However, Table 6 validates that some related security characteristics can be added to each of them. For instance, there are some feasible suggestions:
  - a) Although RBE solved the TTP issue, it requires a PKA, which is a single-point-of-failure for the cryptosystems. Designing a decentralized RBE is a promising approach. The collaboration of a few sub-PKA with together can meet public verifiability, improve decentralization, and keep working without TTP.
  - b) According to the importance of mobility in IoT, designing a BGE that supports dynamic nodes is IoT-friendly. Two TTPs from different BGEs should connect without overshadowing the existing features. In addition, this new cryptosystem can be collusion resistant which prevents any collaboration for corruption.
  - c) Similarly, designing a handover process for connection between two proxies in PRE schemes is a pragmatic approach to back mobile nodes connecting different proxies. A few PRE should accept their re-encrypted messages and do not sacrifice the necessary existing security features.
- In the former section (*Combination*), we mentioned five practical approaches to combine the cryptosystems for jointing their corresponding functionalities. On the combination section, we perform two encryption functions in a row, which is obviously costly. The merging of two advanced cryptosystems to design a unified primitive with features of both sides that holds the properties of both advanced primitives is a promising and practical approach. Therefore, designing an encryption scheme that achieves the characteristics of each combined solution is still five open research directions. For instance, Break-glass PRE/ Break-glass WIE and Proxy BGE/ Proxy WIE might be practical for some IoT-driven scenarios.
- Some IoT scenarios require not only confidentiality driven by encryption schemes but also non-repudiation, unforgeability, and integrity, which are provided by signatures schemes. Signcryption is a cryptographic paradigm that provides the essential properties of both encryption schemes and digital signatures with usually less computation and computation cost than separate signing then encrypting approach [165]. Thus signcryption algorithms are aligned with IoT objectives. Consequently, some innovative IoT-based signcryption schemes can be designed by proficiently combining the conventional or advanced digital signatures (e.g. [166]) with the advanced encryption schemes.
- Although PRE, WIE, RBE, and BGE are computation-wise compared for IoT networks with the classical alternatives, they would be more practical at a lower cost. Thus, the more lightweight versions of the advanced schemes always increase usability in the lower tiers. The future networks will be more efficient and customized to deliver highly aggregated data for real-time analysis by artificai intelligence and federated learning. Thus, efficiency is crucially important in cryptography.

## Conclusion

IoT is comprised of numerous connected and heterogeneous devices to generate and share data. Since the confidentiality of data is crucial, all IoT systems use typical encryption schemes in different layers. However, IoT systems require more functionalities and secure features beyond data confidentiality achieved by some advanced encryption schemes.

This paper is a starting point for the state-of-the-art cryptographic primitives that can be applied in IoT networks. We focused on some new cryptosystems that have not been discussed in the IoT research community yet. First, we thoroughly discussed the cutting-edge technologies in IoT architecture and suggested a multi-tiered IoT architecture based on the edge, fog, cloud, and blockchain technologies. Then, we surveyed and discussed some handpicked, IoT-friendly, and advanced cryptographic primitives, including proxy re-encryption, wildcarded, downgradable, registration-based, and break-glass encryption schemes. Each of these schemes presents a few extra benefits to some parts of IoT systems. This paper can accelerate the development of state-of-the-art cryptography to become prevalent in IoT networks. Many novel security protocols may be designed based on these state-of-the-art primitives. Additionally, there is still much room for improvement in both their theoretical and practical aspects. We extensively discussed the possible approaches for future studies.
